# Bacterial vaginosis associates with dysfunctional T cells and altered soluble immune factors in the cervicovaginal tract

**DOI:** 10.1172/JCI184609

**Published:** 2025-03-25

**Authors:** Finn MacLean, Adino Tesfahun Tsegaye, Jessica B. Graham, Jessica L. Swarts, Sarah C. Vick, Nicole B. Potchen, Irene Cruz Talavera, Lakshmi Warrier, Julien Dubrulle, Lena K. Schroeder, Ayumi Saito, Corinne Mar, Katherine K. Thomas, Matthias Mack, Michelle C. Sabo, Bhavna H. Chohan, Kenneth Ngure, Nelly Rwamba Mugo, Jairam R. Lingappa, Jennifer M. Lund, for the Kinga Study Team

**Affiliations:** 1 Vaccine and Infectious Disease Division, Fred Hutchinson Cancer Center, Seattle, Washington, USA.; 2 Department of Global Health, University of Washington, Seattle, Washington, USA.; 3 Cellular Imaging Shared Resource, Fred Hutchinson Cancer Center. Seattle, Washington, USA.; 4 Department of Internal Medicine–Nephrology, University Hospital Regensburg, Regensburg, Germany.; 5 Department of Medicine, University of Washington, Seattle, Washington, USA.; 6 Center for Virus Research, Kenya Medical Research Institute, Nairobi, Kenya.; 7 School of Public Health, Jomo Kenyatta University of Agriculture and Technology, Nairobi, Kenya.; 8 Center for Clinical Research, Kenya Medical Research Institute, Nairobi, Kenya.; 9 Department of Pediatrics, University of Washington, Seattle, Washington, USA.; 10 See Supplemental Acknowledgments for Kinga Study consortium details.

**Keywords:** AIDS/HIV, Immunology, Infectious disease, T cells

## Abstract

**BACKGROUND:**

Bacterial vaginosis (BV) is a dysbiosis of the vaginal microbiome that is prevalent among reproductive-age females worldwide. Adverse health outcomes associated with BV include an increased risk of sexually acquired HIV, yet the immunological mechanisms underlying this association are not well understood.

**METHODS:**

To investigate BV-driven changes to cervicovaginal tract (CVT) and circulating T cell phenotypes, Kinga Study participants with or without BV provided vaginal tract (VT) and ectocervical (CX) tissue biopsies and PBMC samples.

**RESULTS:**

High-parameter flow cytometry revealed an increased frequency of cervical CD4^+^ conventional T (Tconv) cells expressing CCR5 in BR^+^ versus BR^–^ women. However, we found no difference in the number of CD3^+^CD4^+^CCR5^+^ cells in the CX or VT of BV^+^ versus BV^–^ individuals, suggesting that BV-driven increased HIV susceptibility may not be solely attributed to increased CVT HIV target cell abundance. Flow cytometry also revealed that individuals with BV had an increased frequency of dysfunctional CX and VT CD39^+^ Tconv and CX tissue-resident CD69^+^CD103^+^ Tconv cells, reported to be implicated in HIV acquisition risk and replication. Many soluble immune factor differences in the CVT further support that BV elicits diverse and complex CVT immune alterations.

**CONCLUSION:**

Our comprehensive analysis expands on potential immunological mechanisms that may underlie the adverse health outcomes associated with BV, including increased HIV susceptibility.

**TRIAL REGISTRATION:**

ClinicalTrials.gov NCT03701802.

**FUNDING:**

This work was supported by National Institutes of Health grants R01AI131914, R01AI141435, and R01AI129715.

## Abstract 2

We investigated how bacterial vaginosis affects cervicovaginal T cells to better understand how BV leads to adverse health outcomes including increased risk of HIV susceptibility.

## Introduction

Bacterial vaginosis (BV) is characterized by a vaginal dysbiosis in which the normally *Lactobacillus*-dominated microbiome is replaced by one with higher bacterial diversity, including increased concentrations of anaerobic bacteria (1). BV results in symptoms of vaginal inflammation, discharge, and discomfort, and is estimated to affect 23%–29% of reproductive-age females worldwide (2), making BV the most common cause of vaginal symptoms leading patients to seek medical care (3). BV is also associated with several adverse medical outcomes (1, 2, 4–11), including preterm delivery (12), chorioamnionitis (13), endometritis (14), and an increased risk of HIV acquisition (5, 11). The issue of HIV acquisition risk is of particular concern in parts of the world where both BV and HIV are prevalent, such as in sub-Saharan Africa (15). Although many studies have investigated immune system alterations associated with BV (1, 5–7, 10, 11, 16–19), the link between cervicovaginal tract (CVT) T cells and BV, which may mediate BV-associated adverse health outcomes, is not well understood. Here, we sought to better understand the impact of BV on CVT mucosal and systemic immune responses in Kenyans that may alter HIV susceptibility.

In the CVT, innate and adaptive immune cells line the epithelium and lamina propria to provide immunological surveillance (20). Notably, T cell populations in the CVT maintain distinct phenotypes from those circulating in the blood (21, 22). These include tissue-resident memory T cells (Trms) bearing CD69 with or without CD103 (23). CD69 is a marker of activation (24) that has a unique role in maintaining tissue residency by binding S1PR1 and preventing egress from the tissue (25–27). CD103 further promotes tissue residency by binding to E-cadherin (28). Trms are retained in the mucosal tissue and have the ability to readily respond to pathogens in situ (21, 29–31). Upon recognition of their cognate antigen, Trms use their sensing and alarm function to produce proinflammatory cytokines and chemokines that rapidly recruit and activate other immune cells, thereby potentiating the tissue immune response while also executing their specific effector and cytotoxic functions (32–35).

Given that in the context of heterosexual transmission, genital mucosal tissue is where host exposure to HIV most commonly first occurs, activated CD4^+^ T cells expressing the HIV coreceptor CCR5 in the CVT have been thought to be prime targets for initial HIV infection (36). Studies using a mouse model demonstrated that the introduction of BV-associated bacteria into the vagina led to an increased quantity of CVT CD4^+^ T cells expressing the activation marker CD44 and HIV coreceptor CCR5 (7), suggesting that dysbiosis of normal vaginal flora may lead to an increase of HIV target cells. In humans, studies of immune responses in the context of BV have characterized immune cells collected from the CVT lumen via minimally invasive sampling techniques such as cervicovaginal lavage, vaginal swabs, cervical swabs, or cytobrushes. These studies have identified an increase in CCR5^+^CD4^+^ T cells in the CVT lumen of BV^+^ individuals (37–40), without addressing whether this observation is true in the CVT deeper tissue layers. In addition to the detection of HIV target cells in the CVT lumen of individuals with BV, studies have shown a connection between BV and the presence of proinflammatory cytokines in the CVT, most reliably IL-1β (7, 37, 41, 42), adding further support to the idea that proinflammatory mechanisms underlie the link between BV and increased HIV susceptibility. However, the relationship between BV and an increase in CD4^+^ T cell activation is inconsistently observed (43, 44), suggesting that more complex mechanisms may be contributing to increased HIV susceptibility in individuals with BV. Further characterizing BV-driven immune modulations in the unique immune environment of the CVT is paramount to understanding mechanisms underlying increased HIV susceptibility in individuals with BV.

To comprehensively evaluate immune alterations associated with BV, we used a recent advance in tissue cryopreservation (45) combined with high-parameter, high-throughput flow cytometry to compare immune cells isolated from CVT tissue biopsies and blood from individuals with versus without BV. Given the dense T cell populations within CVT tissue (46), cells isolated from CVT mucosal biopsies may better represent the mucosal tissue immune environment than those isolated from the CVT lumen by cytobrush or other methods. Additionally, improved flow cytometry technology allowed us to broadly evaluate immune cells in the context of BV for phenotypic markers not previously characterized.

Here, we report detailed T cell characteristics of vaginal tract (VT) and ectocervix (CX) mucosal tissues and PBMC samples from the same participants, comparing individuals with versus those without BV. Additionally, we analyzed CVT fluid and serum for local and systemic cytokines and chemokines in individuals with versus without BV. While we did not observe an overall increase in CD3^+^CD4^+^CCR5^+^ HIV target cells in the CVT of BV^+^ individuals, we did observe CX and VT T cells with dysfunctional phenotypes and altered expression of soluble mediators in CVT fluid from BV^+^ individuals. Our findings highlight different mechanisms that may underlie increased HIV susceptibility associated with BV.

## Results

### Study population characteristics.

Two hundred four participants met the criteria to be included in the BV flow cytometry and/or soluble immune factor analysis (Table 1). Forty-six participants contributed at least 1 sample when they were BV^+^ by a Nugent score of 7–10 (34 at study enrollment and 12 at study exit). The BV^–^ comparator group comprised *N* = 158 participants with normal flora (Nugent score 0–3) who provided at least 1 sample at enrollment. Overall, our study population was young (55.9% were <30 years of age) and sexually active (median of 8 unprotected sex acts in the prior month), with 40.8% using hormonal contraceptives and 39.9% herpes simplex virus 2 (HSV-2) seropositive, and 17.6% had a sexual partner living with HIV (Table 1). One hundred eighty-seven participants provided a CX sample (BV^–^ *N* = 149, BV^+^ *N* = 38), 190 participants provided a VT sample (BV^–^ *N* = 149, BV^+^ *N* = 41), and 185 participants provided a PBMC sample (BV^–^ *N* = 143, BV^+^ *N* = 42) for flow cytometry analysis. One hundred ninety-three participants provided a serum sample (BV^–^ *N* = 149, BV^+^ *N* = 44) and 174 participants provided a CVT fluid sample (BV^–^ *N* = 136, BV^+^ *N* = 38) for soluble immune factor analysis. A subset of individuals who were HSV-2 seronegative and unexposed to HIV provided a CX and/or VT tissue sample for immunofluorescent imaging at their enrollment visit (CX BV^–^ *N* = 44, BV^+^ *N* = 11; VT BV^–^ *N* = 55, BV^+^ *N* = 12).

### BV did not alter the balance of T cell frequency in the CVT tissues.

**Figure 1:**
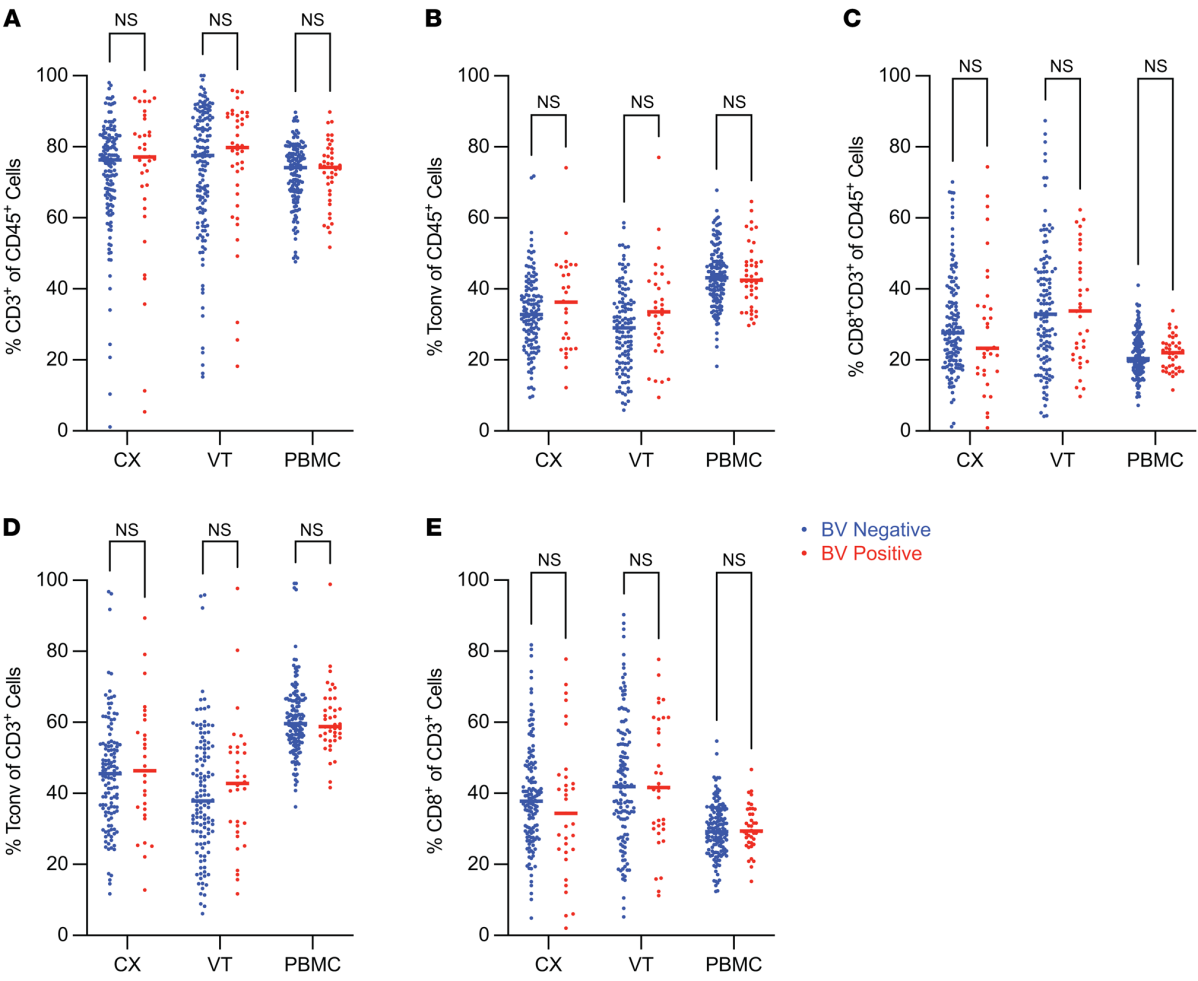
BV did not alter the balance of T cell frequency in the CVT tissues. Flow cytometry was used to quantify the proportions of T cells within different tissue sites as indicated. (**A**) The frequency of CD3^+^ (CX BV^–^ *N* = 147, BV^+^ *N* = 37; VT BV^–^ *N* = 144, BV^+^ *N* = 40; PBMC BV^–^ *N* = 143, BV^+^ *N* = 42) among total CD45^+^ cells. (**B** and **C**) The frequency of Tconv (CX BV^–^ *N* = 129, BV^+^ *N* = 29; VT BV^–^ *N* = 122, BV^+^ *N* = 32; PBMC BV^–^ *N* = 143, BV^+^ *N* = 42) (**B**) and CD3^+^CD8^+^ (CX BV^–^ *N* = 141, BV^+^ *N* = 33; VT BV^–^ *N* = 128, BV^+^ *N* = 36; PBMC BV^–^ *N* = 143, BV^+^ *N* = 42) (**C**) among total CD45^+^ cells. (**D** and **E**) The frequency of Tconv (CX BV^–^ *N* = 129, BV^+^ *N* = 29; VT BV^–^ *N* = 122, BV^+^ *N* = 32; PBMC BV^–^ *N* = 143, BV^+^ *N* = 42) (**D**) and CD8^+^ (CX BV^–^ *N* = 141, BV^+^ *N* = 33; VT BV^–^ *N* = 130, BV^+^ *N* = 36; PBMC BV^–^ *N* = 143, BV^+^ *N* = 42) (**E**) among total CD3^+^ cells. Adjusted rank regression analysis was performed to compare frequencies in each tissue between BV^–^ and BV^+^ individuals. PBMC comparisons were a priori adjusted for hormonal contraceptive use, and CX and VT comparisons were a priori adjusted for hormonal contraceptive use, HSV-2 serology, HIV exposure, and semen exposure, to reduce the effects of potential confounding variables on the analysis of BV-driven T cell alterations. Comparisons with adjusted *P* value > 0.10 labeled as not significant. Each dot represents a measurement from an individual sample. Each horizontal bar indicates the median for its respective group. Additional statistical information for each comparison is provided in Supplemental Table 1.

To investigate the CVT mucosal tissue and circulating T cell population in detail, we performed high-parameter flow cytometry on cells isolated from CX and VT tissue biopsies cryopreserved using a previously published method (45), as well as PBMC samples. PBMC comparisons were a priori adjusted for hormonal contraceptive use, and CX and VT comparisons were a priori adjusted for hormonal contraceptive use, HSV-2 serology, HIV exposure, and semen exposure to reduce the effects of potential confounding variables on the analysis of BV-driven T cell alterations. We found that the majority of CD45^+^ cells in the VT and CX were CD3^+^ T cells (median > 75%; Figure 1A, Supplemental Figure 1, and Supplemental Table 1; supplemental material available online with this article; https://doi.org/10.1172/JCI184609DS1), demonstrating the importance of T cells in immunosurveillance of the CVT. We assessed whether BV was associated with a shift in the proportion of CD25^–^CD127^+/–^ CD4^+^ conventional T (Tconv) cells as a fraction of CD45^+^ cells in all 3 specimen types and found no significant difference comparing BV^–^ versus BV^+^ samples (Figure 1B). The proportion of CD8^+^ T cells was analyzed as a fraction of CD45^+^ cells (Figure 1C), and no significant differences were detected comparing each sample type from BV^–^ versus BV^+^ individuals. The fraction of Tconv cells of total CD3^+^ T cells (Figure 1D) and the fraction of CD8^+^ of total CD3^+^ T cells (Figure 1E) were also not significantly altered by BV for any of the 3 sample types analyzed, indicating that BV may not be associated with a shift in the major T cell subsets.

### Conventional CD4^+^ T cells displayed increased markers of activation and tissue residency in the cervix of individuals with BV.

We hypothesized that BV may be associated with phenotypic alterations among T cell subsets to promote adverse health outcomes. Previous studies have limited the characterization of cervicovaginal T cells to markers of inflammation, including the HIV coreceptor CCR5. Herein, we include markers of activation previously described to be enriched on CD4^+^ T cells of the CVT in the context of persistent BV, including CCR5, HLA-DR, and CD38 (44). However, we additionally sought to broaden our characterization of T cell phenotypic alterations associated with BV. We included Treg lineage markers (CD25, CD127, FoxP3) (47), a Th1 lineage marker (T-bet) (48), Th17 lineage markers (CD161, CCR6) (49, 50), markers of tissue residency (CD69 and CD103) (23), markers of inhibition (CD101 and CTLA-4) (51, 52), dysfunction (CD39) (53), progenitor potential (T cell factor-1 [TCF-1]) (54), exhaustion (PD-1) (55), and an additional chemokine receptor used for trafficking and associated with activation (CXCR3) (56), cytotoxic function (granzyme B) (57), and memory subsets (CCR7 and CD45RA) (58) to more broadly evaluate the phenotypic differences of CVT and circulating T cells in those with versus those without BV.

**Figure 2:**
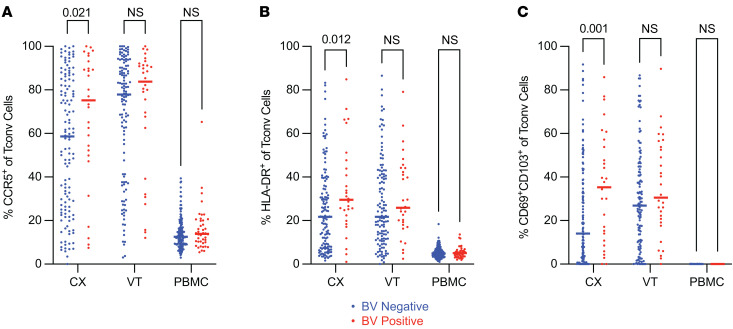
Conventional CD4^+^ T cells displayed increased markers of activation and tissue residency in the cervix of individuals with BV. Flow cytometry was used to examine Tconv cell phenotypes within different tissue sites as indicated. (**A**–**C**) The frequency of CCR5^+^ (**A**), HLA-DR^+^ (**B**), and CD69^+^CD103^+^ (**C**) among total Tconv cells (CX BV^–^ *N* = 128, BV^+^ *N* = 29; VT BV^–^ *N* = 122, BV^+^ *N* = 30; PBMC BV^–^ *N* = 143, BV^+^ *N* = 42). Adjusted rank regression analysis was performed to compare frequencies in each tissue between BV^–^ and BV^+^ individuals. PBMC comparisons were a priori adjusted for hormonal contraceptive use, and CX and VT comparisons were a priori adjusted for hormonal contraceptive use, HSV-2 serology, HIV exposure, and semen exposure, to reduce the effects of potential confounding variables on the analysis of BV-driven T cell alterations. Adjusted *P* value displayed for all *P*_adj_ ≤ 0.10; all other comparisons labeled as not significant. Each dot represents a measurement from an individual sample. Each horizontal bar indicates the median for its respective group. Additional statistical information for each comparison is provided in Supplemental Table 1.

We evaluated whether BV is associated with activated Tconv cell phenotypes that may contribute to increased HIV susceptibility through a previously hypothesized mechanism — CCR5 expression. We found that CX CD4^+^ Tconv cells displayed a higher frequency of the HIV coreceptor CCR5 in BV^+^ compared with BV^–^ individuals (median frequency 59% BV^–^ vs. 75% BV^+^, adjusted *P* value [*P*_adj_] = 0.021; Supplemental Table 1) as detected by flow cytometry and calculated as a fraction of total Tconv cells; minimal differences were observed when VT or PBMC samples from BV^–^ versus BV^+^ individuals were compared (Figure 2A). We also observed a significantly increased frequency of the activation marker HLA-DR on CX Tconv cells in those with versus those without BV (median frequency 22% BV^–^ vs. 30% BV^+^, *P*_adj_ = 0.012; Supplemental Table 1), and minimal differences were observed comparing HLA-DR expression on VT and PBMC Tconv cells from BV^–^ versus BV^+^ individuals (Figure 2B). Together, these results demonstrate that BV does promote local proinflammatory Tconv cell activation and that Tconv cell activation is elevated in the CX mucosa but not in the VT or circulation.

We hypothesized that Trms would play a critical role in immune modulations associated with BV, so we evaluated CD103^+^CD69^+^ coexpression on CD4^+^ Tconv cells as canonical Trm markers (24, 46). We found that the Trm phenotype was more frequently observed on CX Tconv cells in BV^+^ individuals (median frequency 14% BV^–^ vs. 35% BV^+^, *P*_adj_ = 0.001; Supplemental Table 1) and found no significant differences when comparing the frequency of CD103^+^CD69^+^ Tconv cells isolated from VT or PBMC samples from BV^–^ versus BV^+^ individuals (Figure 2C). These results support that BV is associated with an increased proportion of tissue-resident Tconv cells in the CX but not in the VT. Not unexpectedly, Tconv Trms were not present in the circulation.

### The total density of T cells and HIV target cells in the cervix and vagina was not altered by BV.

**Figure 3:**
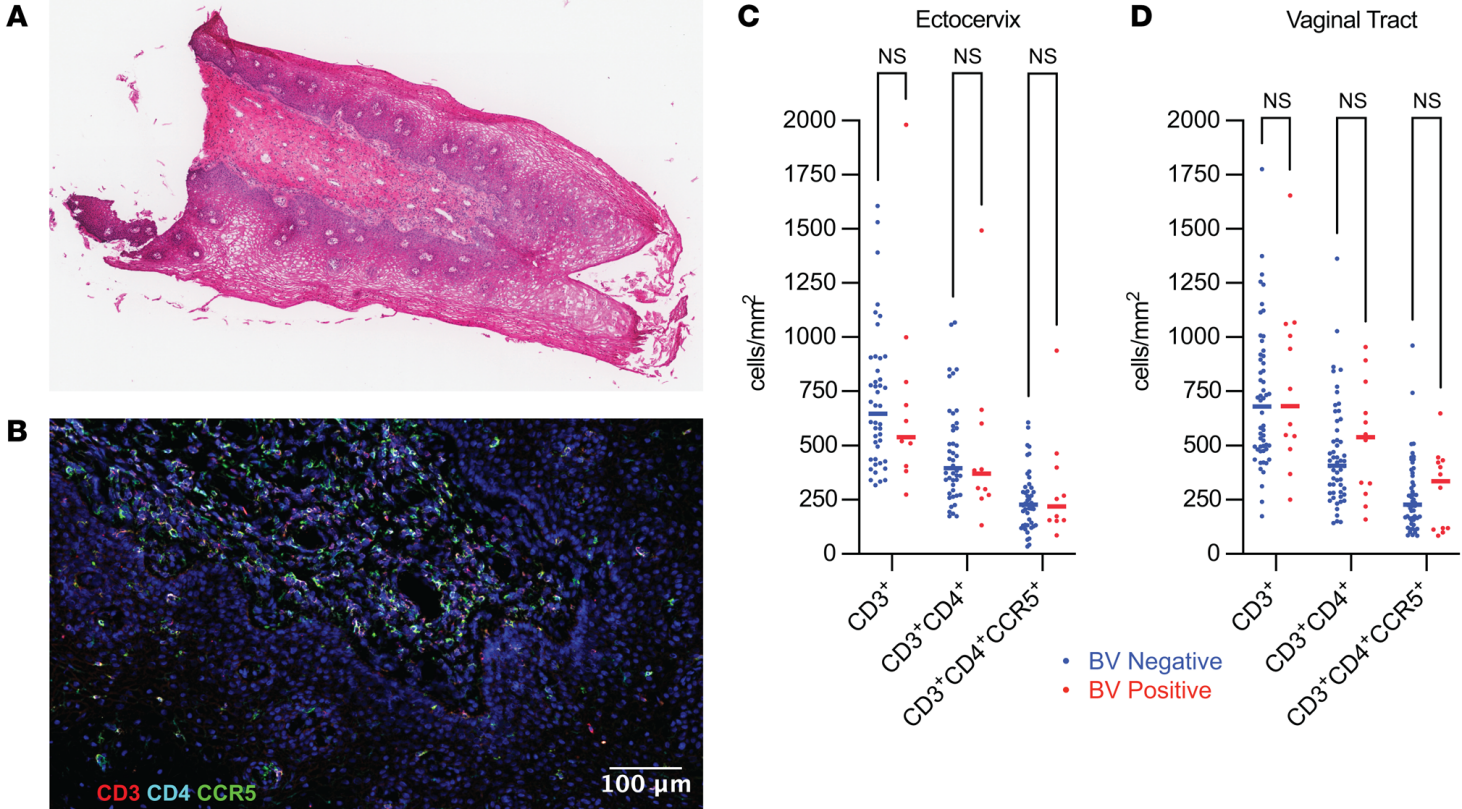
The total density of T cells and HIV target cells in the cervix and vagina was not altered by BV. (**A**) Representative H&E-stained VT tissue section imaged with bright-field microscopy at ×20 magnification. (**B**) Representative immunofluorescently stained tissue section from the same VT biopsy as **A**. DAPI stain is shown in blue, CD3 stain in red, CD4 stain in cyan, and CCR5 stain in green. All fluorescent signals are overlaid. Scale bar: 100 μm. (**C** and **D**) Comparison of the density of CD3^+^ T cells, CD3^+^CD4^+^ T cells, or CD3^+^CD4^+^CD5^+^ HIV target cells in the CX (BV^–^ *N* = 44, BV^+^ *N* = 11) (**C**) and VT (BV^–^ *N* = 55, BV^+^ *N* = 12) (**D**). Wilcoxon’s rank sum test was performed for each comparison shown. Comparisons with *P* > 0.05 labeled as not significant. Each dot represents a measurement from an individual sample. Each horizontal bar indicates the median for its respective group. Additional statistical information for each comparison is provided in Supplemental Table 2.

To assess whether BV is associated with an increased number or density of CCR5^+^ HIV target T cells in the CVT mucosa, we analyzed VT and CX tissue sections by immunofluorescent staining and microscopy. H&E staining was used to evaluate tissue quality and integrity and visualize the epithelium and lamina propria compartments (representative image, Figure 3A). Immunofluorescent staining and cellular imaging were used to visualize CD3^+^, CD3^+^CD4^+^, and CD3^+^CD4^+^CCR5^+^ cells in each tissue section (representative image, Figure 3B). In contrast to the increased frequency of CCR5^+^ Tconv cells that we observed as a fraction of total Tconv cells in the CX by flow cytometry (Figure 2A), we found no significant differences in the overall density of HIV target T cells in the CX (Figure 3C, median density 228.3 cells/mm^2^ BV^–^ vs. 219.6 cells/mm^2^ BV^+^, *P* = 0.764; Supplemental Table 2) or VT (Figure 3D, median density 227.0 cells/mm^2^ BV^–^ vs. 336.2 cells/mm^2^ BV^+^, *P* = 0.787; Supplemental Table 2). We also observed no difference when analyzing epithelium and lamina propria tissue compartments individually (Supplemental Figure 2 and Supplemental Table 2), suggesting that BV may not significantly alter the overall abundance of HIV target cells within the deeper CX or VT mucosal tissue layers.

### Conventional CD4^+^ T cells in the cervix showed signs of dysfunction in individuals with BV.

**Figure 4:**
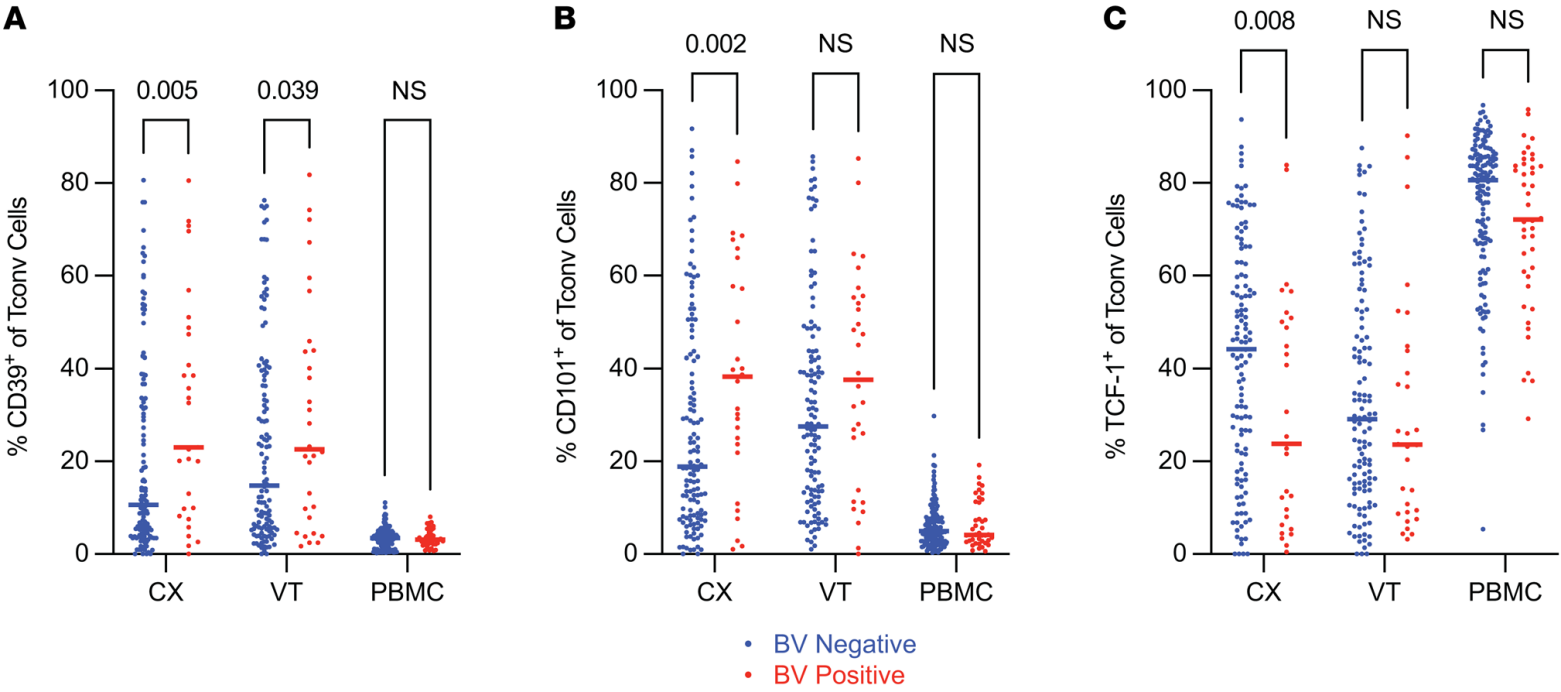
Conventional CD4^+^ T cells in the cervix showed signs of dysfunction in individuals with BV. Flow cytometry was used to examine Tconv cell phenotypes within different tissue sites as indicated. (**A**–**C**) The frequency of CD39^+^ (**A**), CD101^+^ (**B**), and T cell factor-1^+^ (TCF-1^+^) (**C**) among total Tconv cells (CX BV^–^ *N* = 128, BV^+^ *N* = 29; VT BV^–^ *N* = 122, BV^+^ *N* = 30; PBMC BV^–^ *N* = 143, BV^+^ *N* = 42). Adjusted rank regression analysis was performed to compare frequencies in each tissue between BV^–^ and BV^+^ individuals. PBMC comparisons were a priori adjusted for hormonal contraceptive use, and CX and VT comparisons were a priori adjusted for hormonal contraceptive use, HSV-2 serology, HIV exposure, and semen exposure, to reduce the effects of potential confounding variables on the analysis of BV-driven T cell alterations. Adjusted *P* value displayed for all *P*_adj_ ≤ 0.10; all other comparisons labeled as not significant. Each dot represents a measurement from an individual sample. Each horizontal bar indicates the median for its respective group. Additional statistical information for each comparison is provided in Supplemental Table 1.

To more broadly characterize how BV impacts mucosal or systemic T cells that may elucidate alternative immunological mechanisms for adverse outcomes associated with BV, we evaluated Tconv cells for additional markers that could indicate altered functional capacity. We found CD39 expression significantly increased on Tconv cells in CX samples from BV^+^ versus BV^–^ individuals (median frequency 11% BV^–^ vs. 23% BV^+^, *P*_adj_ = 0.005; Supplemental Table 1) and in VT samples from BV^+^ versus BV^–^ individuals (median frequency 15% BV^–^ vs. 23% BV^+^, *P*_adj_ = 0.039; Supplemental Table 1), with minimal differences observed in PBMC samples (Figure 4A). CD39 is a marker of metabolic stress (53) that may contribute to Tconv cell dysfunction in the CX and VT of BV^+^ individuals. CD101 expression was also significantly increased on Tconv cells in CX samples from BV^+^ versus BV^–^ individuals (median frequency 19% BV^–^ vs. 38% BV^+^, *P*_adj_ = 0.002; Supplemental Table 1), with minimal differences observed in VT and PBMC samples (Figure 4B). CD101 is another marker associated with tissue residency (23), though it has also been shown to contribute to an inhibitory phenotype (51) that may impede the proliferation of Tconv cells in BV^+^ individuals. Finally, we found a significant decrease in the frequency of TCF-1^+^ Tconv cells in the CX of BV^+^ compared with BV^–^ individuals (median frequency 44% BV^–^ vs. 24% BV^+^, *P*_adj_ = 0.008; Supplemental Table 1), with no significant differences in the VT or PBMCs (Figure 4C). TCF-1 is a stabilizing transcription factor implicated in the regulation of progenitor potential and promotion of T cell fate specification (54, 59–64). These results suggest that Tconv cells in the CX of BV^+^ individuals may have a reduced capacity to undergo differentiation or serve as proliferative progenitor cells, which, taken together, could suggest that these cells have reduced capacity to serve as potent effector T cells.

### Th17 cells exhibited increased markers of activation and tissue residency in cervical samples from individuals with BV.

**Figure 5:**
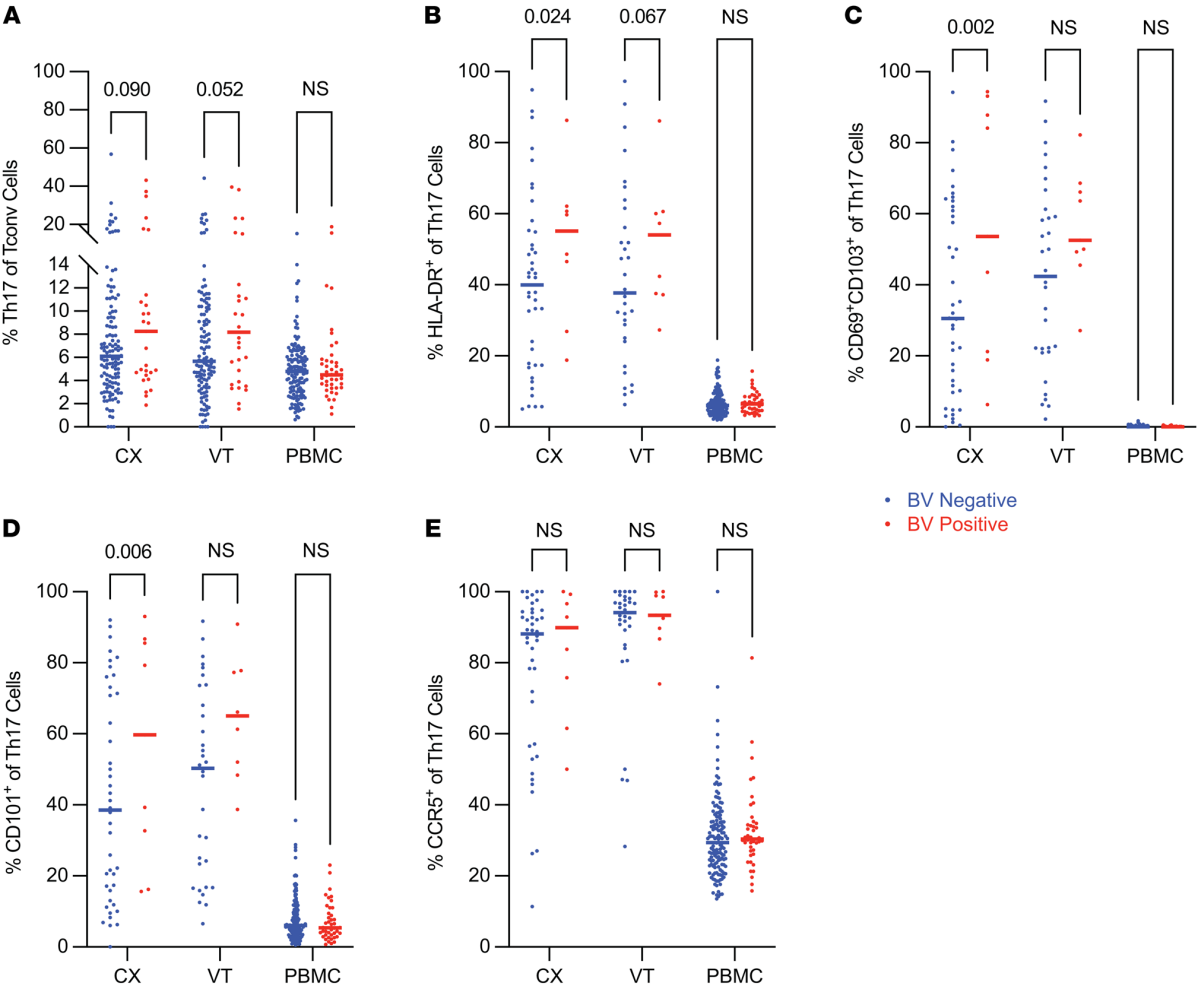
Th17 cells exhibited increased markers of activation and tissue residency in cervical samples from individuals with BV. Flow cytometry was used to examine Th17 phenotypes within different tissue sites as indicated. (**A**) The frequency of Th17 cells, defined as CD161^+^CCR6^+^ Tconv, among total Tconv cells (CX BV^–^ *N* = 128, BV^+^ *N* = 29; VT BV^–^ *N* = 122, BV^+^ *N* = 30; PBMC BV^–^ *N* = 143, BV^+^ *N* = 42). (**B**–**E**) HLA-DR^+^ (**B**), CD69^+^CD103^+^ (**C**), CD101^+^ (**D**), and CCR5^+^ (**E**) frequencies among total Th17 cells (CX BV^–^ *N* = 42, BV^+^ *N* = 9; VT BV^–^ *N* = 32, BV^+^ *N* = 9; PBMC BV^–^ *N* = 143, BV^+^ *N* = 42). Adjusted rank regression analysis was performed to compare frequencies in each tissue between BV^–^ and BV^+^ individuals. PBMC comparisons were a priori adjusted for hormonal contraceptive use, and CX and VT comparisons were a priori adjusted for hormonal contraceptive use, HSV-2 serology, HIV exposure, and semen exposure, to reduce the effects of potential confounding variables on the analysis of BV-driven T cell alterations. Adjusted *P* value displayed for all *P*_adj_ ≤ 0.10; all other comparisons labeled as not significant. Each dot represents a measurement from an individual sample. Each horizontal bar indicates the median for its respective group. Additional statistical information for each comparison is provided in Supplemental Table 1.

Lineage-specific Th17 Tconv (Th17) cells have been shown to play a role in immune responses to extracellular bacteria (65–67). To investigate the impact of BV on Th17 cells, we first analyzed the frequency of Th17 cells (defined as CD161^+^CCR6^+^ Tconv cells; ref. 49) (Supplemental Figure 1) among total Tconv cells and found that the frequency of Th17 cells as a proportion of total Tconv cells was not significantly increased in any of the tissue types comparing BV^+^ and BV^–^ individuals (Figure 5A). However, the frequency of Th17 cells expressing the proinflammatory activation marker HLA-DR was significantly increased in CX during BV (median 40% BV^–^ vs. 55% BV^+^, *P*_adj_ = 0.024; Supplemental Table 1) with a trend toward an increase in the VT during BV (median 38% BV^–^ vs. 54% BV^+^, *P*_adj_ = 0.067; Supplemental Table 1) and minimal differences in PBMCs from BV^–^ versus BV^+^ individuals (Figure 5B).

We also measured the frequency of markers of tissue residency (CD69^+^CD103^+^) on Th17 cells and found that there was a significant increase in the fraction of Th17 cells that are CD69^+^CD103^+^ in the CX of BV^+^ compared with BV^–^ individuals (median 31% BV^–^ vs. 54% BV^+^, *P*_adj_ = 0.002; Supplemental Table 1) (Figure 5C). In addition, the frequency of Th17 cells expressing CD101, consistent with a Trm phenotype, was significantly increased in the CX of BV^+^ compared with BV^–^ individuals (median 39% BV^–^ vs. 60% BV^+^, *P*_adj_ = 0.006; Supplemental Table 1) (Figure 5D).

Finally, we found that the frequency of Th17 cells expressing the activation marker and HIV coreceptor CCR5 was not significantly different between BV^–^ versus BV^+^ individuals, although, notably, the median frequency of Th17 cells expressing CCR5 in BV^+^ individuals was 90% in CX and 93% in VT samples (Supplemental Table 1 and Figure 5E). Therefore, CVT Th17 cells predominantly express the HIV coreceptor CCR5, consistent with previous reports that Th17 cells are viable targets for HIV infection (68). These results demonstrate that BV is associated with CX Th17 activation and that the increased tissue-resident or CD101-expressing CX Th17 cells may contribute to a mechanism for increased HIV susceptibility in individuals with BV.

### Cervical CD8^+^ T cells displayed a dysfunctional phenotype in individuals with BV.

**Figure 6:**
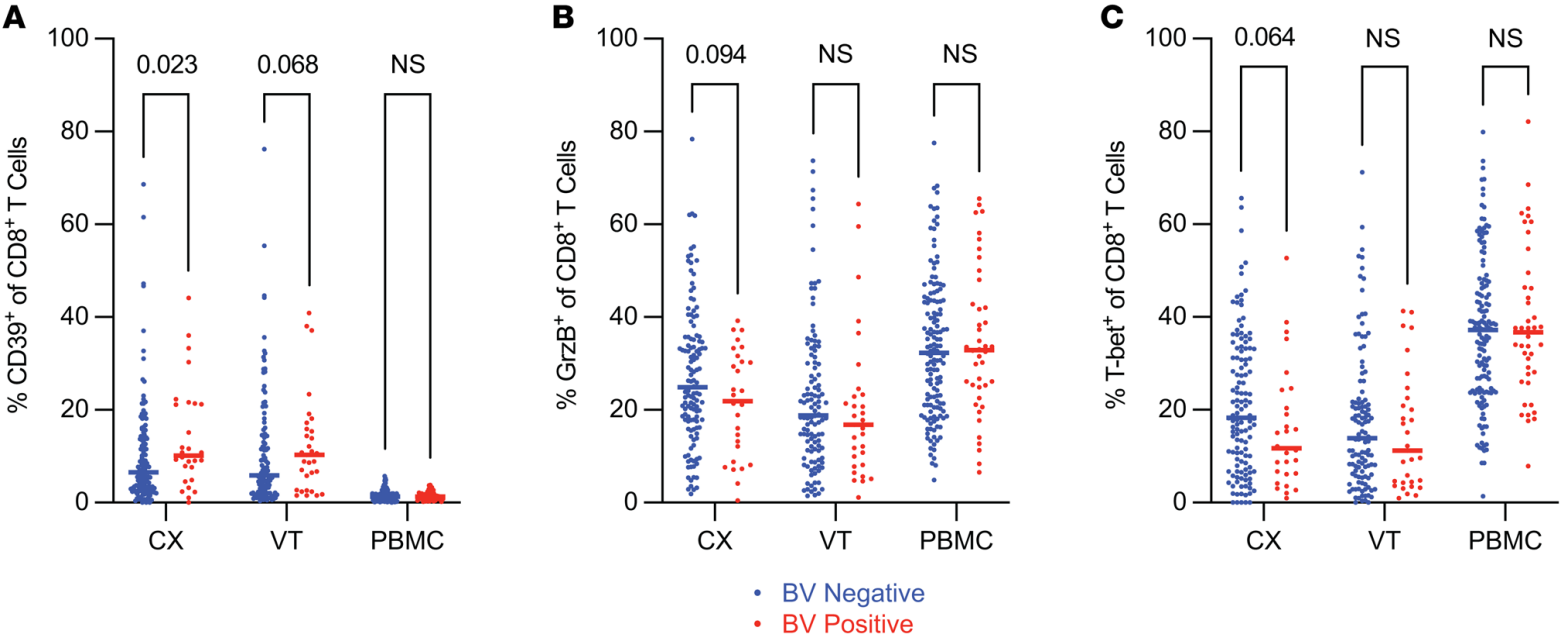
Cervical CD8^+^ T cells displayed a dysfunctional phenotype in individuals with BV. Flow cytometry was used to examine CD8^+^ T cell phenotypes within different tissue sites as indicated. (**A**–**C**) The frequency of CD39^+^ (**A**), granzyme B^+^ (**B**), and T-bet^+^ (**C**) among total CD8^+^ T cells (CX BV^–^ *N* = 127, BV^+^ *N* = 29; VT BV^–^ *N* = 116, BV^+^ *N* = 31; PBMC BV^–^ *N* = 143, BV^+^ *N* = 42). Adjusted rank regression analysis was performed to compare frequencies in each tissue between BV^–^ and BV^+^ individuals. PBMC comparisons were a priori adjusted for hormonal contraceptive use, and CX and VT comparisons were a priori adjusted for hormonal contraceptive use, HSV-2 serology, HIV exposure, and semen exposure, to reduce the effects of potential confounding variables on the analysis of BV-driven T cell alterations. Adjusted *P* value displayed for all *P*_adj_ ≤ 0.10; all other comparisons labeled as not significant. Each dot represents a measurement from an individual sample. Each horizontal bar indicates the median for its respective group. Additional statistical information for each comparison is provided in Supplemental Table 1.

CD8^+^ T cells are thought to play an important role in pathogen clearance in the CVT, including against BV-associated organisms. To comprehensively evaluate how BV impacts CD8^+^ T cell phenotypes in the CVT mucosa and circulation, we analyzed CX, VT, and PBMC samples for the expression of effector markers (Supplemental Figure 1 and Supplemental Table 1). We found that the frequency of CD39^+^CD8^+^ T cells was significantly increased in CX samples from BV^+^ versus BV^–^ individuals (median frequency 7% BV^–^ vs. 10% BV^+^, *P*_adj_ = 0.023; Supplemental Table 1), and moderately but insignificantly increased in the VT of BV^+^ individuals (median frequency 6% BV^–^ vs. 10% BV^+^, *P*_adj_ = 0.068; Supplemental Table 1 and Figure 6A). We also observed a moderate though insignificant decrease in granzyme B expression, a marker of cytotoxicity, on CD8^+^ T cells in the CX of BV^+^ versus BV^–^ individuals (median frequency 25% BV^–^ vs. 22% BV^+^, *P*_adj_ = 0.094; Supplemental Table 1), with marginal differences in the VT and PBMC samples from those with versus those without BV (Figure 6B). Additionally, we observed a moderate but insignificant decrease in the frequency of T-bet^+^ CD8^+^ T cells in the CX of BV^+^ versus BV^–^ individuals (median frequency 18% BV^–^ vs. 12% BV^+^, *P*_adj_ = 0.064; Supplemental Table 1), with marginal differences in the VT and PBMC samples from those with versus those without BV (Figure 6C). Altogether, these findings suggest a trend toward decreased CX CD8^+^ T cell activation in addition to an increased dysfunctional CD39^+^ CD8^+^ T cell phenotype observed in the CX of BV^+^ individuals.

### BV was associated with reduced chemokine concentrations and increased inflammatory cytokine concentrations in cervicovaginal fluid.

**Figure 7:**
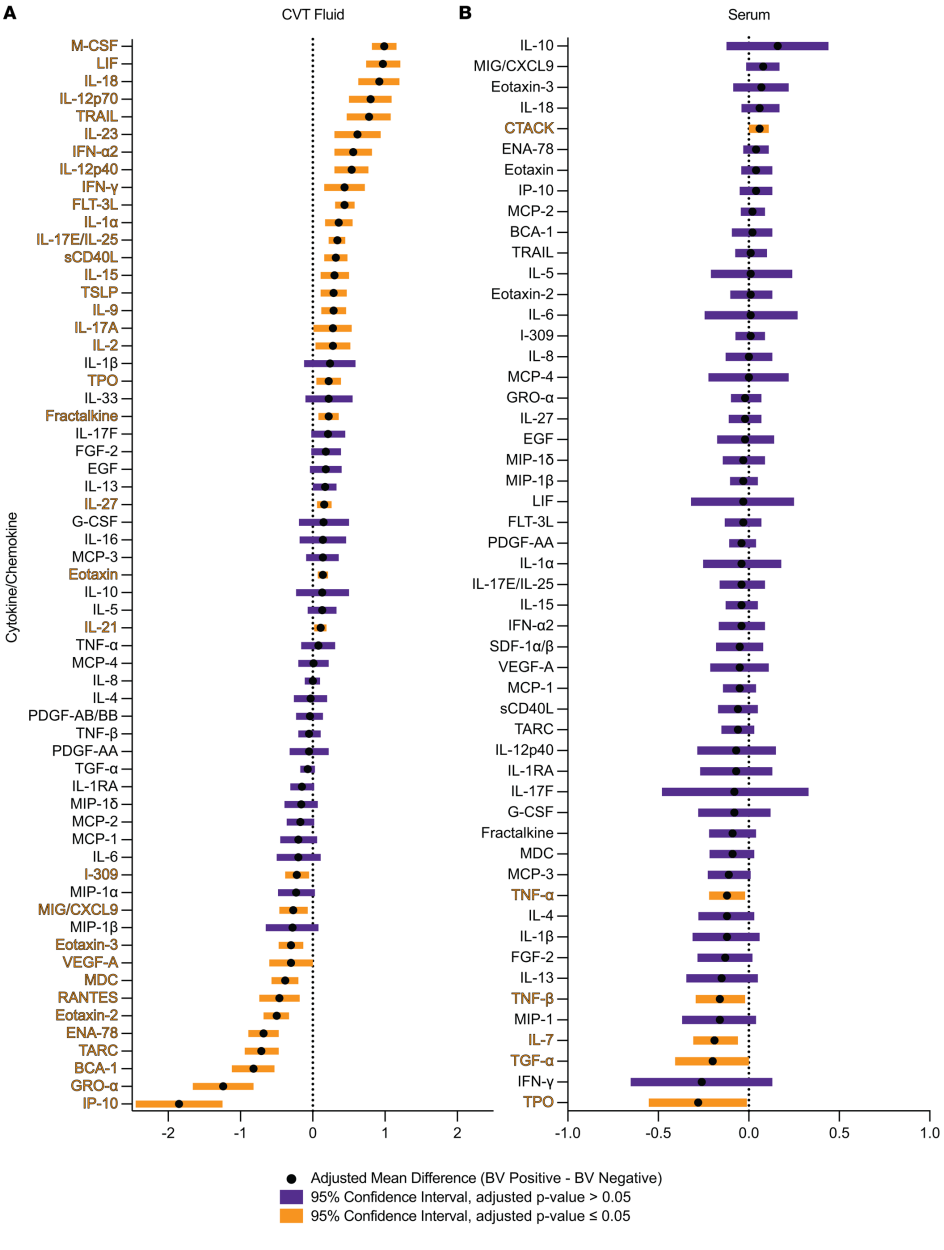
BV was associated with reduced chemokine concentrations and increased inflammatory cytokine concentrations in CVT fluid. Luminex was used to quantify cytokines and chemokines from CVT fluid (BV^–^ *N* = 134, BV^+^ *N* = 36 for IL-4; BV^–^ *N* = 135, BV^+^ *N* = 37 for IL-2; BV^–^ *N* = 136, BV^+^ *N* = 37 for LIF and TARC; BV^–^ *N* = 136, BV^+^ *N* = 38 for all other cytokine/chemokine comparisons) (**A**) and serum (BV^–^ *N* = 149, BV^+^ *N* = 44) (**B**). Adjusted estimated mean difference (BV^+^ – BV^–^) for CVT fluid cytokines/chemokines and serum cytokines/chemokines that met the criteria for quantification of cytokine/chemokine concentrations (≥80% of samples were detectable). The adjusted 95% confidence interval is shown for all comparisons. Significant results when *P*_adj_ ≤ 0.05 comparing BV^–^ versus BV^+^ are colored in orange, and non-significant differences (*P* > 0.05) comparing BV^–^ versus BV^+^ are purple. Vertical dashed line at *x* = 0 for reference. Serum comparisons were a priori adjusted for hormonal contraceptive use, and CVT fluid comparisons were a priori adjusted for hormonal contraceptive use, HSV-2 serology, HIV exposure, and semen exposure, to reduce the effects of potential confounding variables on the analysis of BV-driven T cell alterations. Additional statistical information for each comparison is provided in Supplemental Table 3.

To investigate soluble immune factors in individuals with BV versus those without BV, we analyzed 71 cytokines and chemokines in serum and CVT fluid collected via menstrual cup (Softcup). In CVT fluid samples, 61 cytokines and chemokines met the criteria for continuous analysis (Figure 7A), while 10 cytokines and chemokines were analyzed as a dichotomous outcome (detected/not detected; Supplemental Figure 3A). In the serum, 52 different cytokines and chemokines met the criteria for continuous analysis (Figure 7B), 17 cytokines and chemokines were analyzed using the dichotomous outcome (Supplemental Figure 3B), and 2 cytokines were detectable in all individuals but had greater than 20% of samples out of range high and so were excluded from the analysis (Supplemental Table 3). We observed significantly higher concentrations of 23 soluble immune factors, including IL-1α, in CVT fluid collected from participants with versus without BV (Figure 7A). Additionally, we observed significantly lower concentrations of 12 soluble immune factors, including MIG/CXCL9 and IP-10 (CXCL10) (Figure 7A), in participants with BV versus those without BV. In our analysis of serum cytokines and chemokines, fewer significant results were observed than in the CVT fluid. In total, we observed a significant increase of 1 and a significant reduction of 5 quantifiable soluble immune factors in participants with versus without BV (Figure 7B). In the CVT fluid samples, of the 10 soluble immune factors analyzed for their dichotomous outcome, 3 were detected significantly more frequently and 1 cytokine was detected significantly less frequently in BV^+^ individuals (Supplemental Figure 3A). The dichotomous outcome analysis revealed an additional 2 other cytokines observed significantly less frequently in the serum of BV^+^ individuals (Supplemental Figure 3B). Overall, differentially expressed and detected soluble immune factors were primarily observed in CVT fluid samples, with fewer differences observed in serum samples, highlighting that BV has the greatest effects on the local CVT, as opposed to the circulating immune environment.

## Discussion

In the context of the global HIV pandemic, T cells serve as a double-edged sword, mediating antiviral functions required to fight the virus while also acting as potential targets for HIV infection and replication (69, 70). The findings presented here add further detail that may underlie the effect of BV on HIV susceptibility by highlighting a more complex scenario that involves deleterious effects on T cell antiviral functions in addition to modulations in subsets that may act as potential HIV target cells.

Our flow cytometry analysis revealed an increase in the frequency of activation markers expressed on cervical Tconv cells, including a higher frequency of CCR5^+^ among total Tconv cells (Figure 2A), which supports prior studies that have identified increased levels of total CD3^+^CD4^+^CCR5^+^ T cells in the CVT lumen as a potential source of HIV target cells (7, 39, 40). However, our analysis of CD3^+^CD4^+^CCR5^+^ HIV target cell tissue density by immunofluorescent staining and cellular imaging of tissue sections indicated a relatively equivalent abundance of CD3^+^CD4^+^CCR5^+^ HIV target cells among BV^–^ and BV^+^ participants in CX and VT tissue evaluated overall (Figure 3, C and D) and when the epithelium and lamina propria compartments were analyzed separately (Supplemental Figure 2). This supports the idea that BV does not have a profound effect on the overall abundance of HIV target cells in the cervicovaginal mucosa. Our conclusions are limited by the fact that the results are based in distinct technologies (flow cytometry versus microscopy), and that flow cytometry involves the evaluation of more samples and therefore has increased power to detect differences compared with microscopy. Nevertheless, our results underscore that the abundance of CD3^+^CD4^+^CCR5^+^ HIV target cells within the deeper CX and VT tissue layers in BV^+^ individuals is unlikely to solely account for increased HIV susceptibility among those with BV, and this raises the possibility that other factors contribute to this adverse outcome.

Our application of high-parameter, high-throughput flow cytometry on cells isolated from VT and CX tissue biopsies and PBMC samples documents that BV elicits complex changes to CVT T cells that may contribute to the inhibition of antiviral T cell function. We observed an increased frequency of CD4^+^ Tconv cells expressing CD39 in the CX and VT of BV^+^ individuals (Figure 4, A and B). The potential dysfunction of this T cell phenotype is suggested by prior studies showing that CD39^+^ T cells have a decreased response to vaccines and an increased likelihood of undergoing apoptosis (53), raising the possibility that in the context of BV, cervical and vaginal CD4^+^ Tconv cells may exhibit increased intrinsic immunoregulation leading to reduced effector potential. This could lead to increased HIV susceptibility through an inability to mount an effective antiviral response upon exposure to the virus.

We also found that CD101^+^ Tconv cells, and specifically CD101^+^ Th17 cells, are more frequently observed in the CX of BV^+^ individuals (Figure 4B and Figure 5D). Less is known about the function of CD101^+^ T cells in comparison with the other phenotypes examined in this study, but CD101^+^ T cells have been associated with decreased expansion after adoptive transfer in mice (51), suggesting that CD101^+^ Tconv cells are inhibited and less likely to undergo proliferation. Additionally, CD101 is associated with exhausted T cell phenotypes that result from chronic activation and can promote immune dysfunction (71–73). Thus, the increased abundance of CD101^+^ Tconv and Th17 cells in the CX of BV^+^ individuals may also contribute to a dysfunctional Tconv cell immune response upon secondary exposure to HIV in the CX. Given the often recurrent and persistent nature of BV (44), chronic immune activation from repeated antigen exposure caused by BV-associated microbiota may lead to the progression of these observed dysfunctional phenotypes that could promote HIV susceptibility by limiting antiviral T cell function, expansion, and differentiation.

We also observed a significant reduction in the proportion of TCF-1^+^ CD4^+^ Tconv cells in the CX of BV^+^ individuals (Figure 4C). TCF-1 is necessary for follicular T cell differentiation (59–61) and has been shown to play a role in Th17 responses by inhibiting IL-17 production (64). Fewer progenitor Tconv cells implies that more Tconv cells are likely to be terminally differentiated in the CX, which may further limit lineage-specific differentiation upon secondary exposure to an infectious agent.

In addition to CD4^+^ T cells expressing CCR5, it has been shown that CD69^+^CD4^+^ T cells are associated with increased susceptibility to HIV infection in the CVT mucosal tissue (74). This suggests that the increased frequency of Tconv Trms in the CX of BV^+^ individuals (Figure 2C) may mediate increased HIV acquisition in the context of BV. In particular, we found that the CX of those with BV had an increased frequency of Th17 Trms (Figure 5C), which highly express CCR5 (Figure 5E); thus, these cells are potential targets for HIV infection. The possibility that these cells are a target for HIV acquisition in BV^+^ individuals is supported by a prior report that Th17 cells serve as primary target cells during vaginal SIV infection (75). In addition to the role CD4^+^ Trms in CVT mucosa play in HIV susceptibility, they also act as reservoirs for HIV viral replication, which can occur during early HIV infection (76). The increased frequency of Tconv Trms, and in particular Th17 Trms, may not only contribute to susceptibility to primary HIV infection, but may also promote viral replication in the CX of BV^+^ individuals.

Activated CD8^+^ T cells are thought to be important in controlling HIV replication after initial infection (77, 78). The increased frequency of mucosal CD39^+^ CD8^+^ T cells observed in individuals with BV may also augment CVT immune dysfunction. Trends toward decreased CX granzyme B^+^ or T-bet^+^ CD8^+^ T cell activation (Figure 6, B and C) may relate to the increased frequency of dysfunctional CD4^+^ Tconv cells observed (Figures 4 and 5), since functional CD4^+^ Tconv cells are known to enhance CD8^+^ T cell effector function (79), and in the context of BV, CD4^+^ Tconv cells appear to be diminished in function. The interactions, or possibly lack thereof, between CD8^+^ T cells and dysfunctional Tconv cells in the context of BV may also contribute to a dysfunctional CD8^+^ T cell response during BV and increased HIV susceptibility in BV^+^ individuals by inhibiting an effective CD8^+^ T cell response after exposure to HIV.

Our analysis of soluble immune factor detection in BV^+^ versus BV^–^ individuals further demonstrates the diverse immune modulations that BV can promote in the CVT. We observed significant increases in several proinflammatory cytokines in BV^+^ CVT fluid samples, including IL-1α, as previously reported by other studies (80–82). We also observed significantly increased IL-17A, IFN-γ, IL-21, IL-23, and IL-12p70 in the CVT fluid of BV^+^ individuals (Figure 7A), all of which can enhance Th17-mediated inflammation (49, 66, 83–85). The activation of Th17 cells may then lead to the formation of Th17 Trms, which were observed more frequently in the CX of BV^+^ individuals by flow cytometry (Figure 5C). Additionally, increased M-CSF in the CVT fluid of BV^+^ individuals (Figure 7A), produced by macrophages, has been shown to promote Th17 differentiation of CD4^+^ memory T cells (86), which may also contribute to the expanded Th17 Trm pool in the CX of BV^+^ individuals. On the other hand, LIF and IL-17E/IL-25, observed to be increased in the CVT fluid of BV^+^ individuals (Figure 7A), inhibit inflammatory responses, including Th17-mediated immune responses (87, 88). LIF and IL-17E/IL-25 may be produced in response to the activation of Th17 cells to limit overall Th17 cell differentiation and proliferation. The reduction of TCF-1^+^ progenitor Tconv cells concurrent with the production of LIF and IL-17E/IL-25 in the CVT could limit the differentiation of progenitor T cells to Th17 cells, which could explain why we do not observe a significant overall expansion in frequency of Th17 cells in BV^+^ individuals (Figure 5A).

While we observe an increased concentration of many proinflammatory cytokines in BV^+^ CVT fluid samples, we also observe the reduction of many T cell–recruiting chemokines. Our results support previous studies that have also observed reductions of IP-10 (CXCL10) and MIG/CXCL9 in the CVT of BV^+^ individuals (82, 89–91). We hypothesized that RANTES (CCL5) would be increased in BV^+^ individuals (37) to promote the recruitment of CCR5^+^ HIV target cells to the CVT. However, our observed reduction of RANTES in the CVT fluid of BV^+^ individuals has also been reported in a prior study that evaluated CVT fluid collected by menstrual cup (90). While RANTES does act as a primary ligand for CCR5, which can promote the recruitment of CCR5^+^ cells, it also reduces cell-surface expression of CCR5 by binding and internalizing the CCR5 receptor (92). Increased CVT RANTES has been associated with increased HIV resistance (93), possibly by competing with HIV for CCR5 binding (94). Reduced RANTES in BV^+^ individuals may limit the number of CCR5^+^ HIV target cells that are recruited to the CVT. Simultaneously, this could promote stabilization of CCR5 expression on existing T cells in the CVT by reducing ligand availability and binding to internalize residually expressed CCR5, a protein preferentially observed on CVT Tconv cells versus circulating Tconv cells (Figure 2A). The relationship between CCR5 expression and CVT RANTES production in BV^+^ individuals is complex, and how these factors impact HIV susceptibility over time will require more detailed longitudinal observational studies. The reduction of IP-10, MIG/CXCL9, RANTES, and several other proinflammatory chemokines in the CVT fluid of BV^+^ individuals supports the notion of impaired T cell recruitment to the CVT, which adds further evidence to dysregulated CVT immune responses in BV^+^ individuals. This may also contribute to adverse health outcomes, including increased HIV susceptibility, by inhibiting antiviral T cell recruitment to the CVT in BV^+^ individuals.

In addition to our a priori hypothesis of an increase in RANTES in the CVT fluid of BV^+^ individuals, we also hypothesized that IL-6, IL-8, and IL-1β would each be increased in BV^+^ individuals based on a previous meta-analysis (37). We observed no significant difference in IL-6, IL-8, or IL-1β in CVT samples from BV^+^ versus BV^–^ individuals. However, CVT IL-1β was trending toward an increase in BV^+^ (adjusted log mean difference BV^+^ – BV^–^ = 0.24; *P*_adj_ = 0.188; Figure 7A and Supplemental Table 3). While IL-1β is frequently described as increased in CVT fluid from BV^+^ individuals, this is not a universal observation (95, 96), nor is IL-6 or IL-8 uniformly increased across cohorts of BV^+^ individuals (97, 98). The factors that mediate altered expression of soluble immune mediators during BV have not been elucidated, highlighting the complexity and variability of BV-driven CVT immune responses.

A majority of the significant phenotypic alterations observed in this study occurred when CX samples from individuals with versus without BV were compared. This indicates that while BV is a dysbiosis of vaginal flora, the immune cells in the CX may be more greatly impacted by exposure to BV-associated organisms than the immune cells in the VT. One reason for this may be that the unique CX microbiota (99) may be more sensitive to changes in flora than the VT, or that the immune cells of the VT may be more resistant to BV-driven phenotypic alterations than those in the CX. The primary cellular targets for intravaginal infection of SIV in rhesus macaques are in the CX lamina propria (100), suggesting that the CX may be the primary site of sexual transmission of HIV in humans. Therefore, the more distinct immune alterations observed in the CX of BV^+^ individuals may be of particular concern for the increased risk of HIV transmission and replication in BV^+^ individuals. Comparison of immune modulations in the CX versus VT among those with or without BV warrants further exploration.

Our study has several limitations. First, we focused on characterizing diverse T cell markers and a large panel of soluble immune mediators in the context of BV, leaving the relationship of BV to other components of the immune response (monocytes/macrophages, granulocytes, and other immune cell types) unevaluated. One justification for limiting our evaluation to characteristics of CD3^+^ T cells is that these constitute the predominant lymphocyte population in the CVT mucosa (Figure 1A). A second limitation is that we collected 3-mm tissue biopsies that often captured low amounts of rare T cell subsets such as Tregs. We were underpowered to evaluate CX Treg phenotypes (BV^+^ *N* = 2) or VT Treg phenotypes (BV^+^ *N* = 4) (Supporting Data Values file). This also reduced our ability to effectively evaluate the role of other rare subsets in immune responses to BV. Third, an alternative hypothesis for adverse health outcomes associated with BV is that BV drives epithelial damage, which, in the context of HIV susceptibility, reduces the effectiveness of the epithelial barrier in providing a physical barrier to protect against HIV transmission. We attempted to quantify tissue damage (data not shown) but were limited by sample quality for blinded pathology scoring. A fourth limitation to our study was that tissue biopsies were variable in size, so we could not compare cell numbers in tissue by flow cytometry. Future studies will include weighing of tissue biopsies before cryopreservation to normalize the number of cells analyzed by flow cytometry per tissue biopsy by weight. A final limitation of our analysis is that it was performed as an exploratory, hypothesis-generating analysis and, as such, used nominal *P* values adjusted for confounders but not discounted for multiple comparisons (138 immune cell subset comparisons, and 71 soluble mediator comparisons). Thus, our findings should be confirmed through studies employing hypothesis-driven testing.

In summary, our study performed the most comprehensive evaluation of CVT tissue T cell subsets associated with BV to date. These data show that BV-driven immune alterations have the most profound impact on CX T cells and changes at this site may be most responsible for increased HIV susceptibility in BV^+^ individuals. While we observe increased CX Tconv cell activation, including increased frequency of CCR5 detection on CX Tconv cells by flow cytometry, our analysis of CX and VT tissue sections by immunofluorescent microscopy shows that BV does not have a profound effect on overall CCR5^+^ HIV target cell density. Our high-parameter, high-throughput flow cytometry analysis revealed that BV drives diverse phenotypic alterations that extend beyond CD4^+^ Tconv cell activation in the CVT. Specifically, we identify an increased frequency of dysfunctional T cell subsets, including CD39^+^ Tconv and CD39^+^ CD8^+^ T cells, and a decreased frequency of progenitor TCF-1^+^ Tconv cells in the CX of BV^+^ individuals that could alter host response and contribute to increased HIV susceptibility by limiting the antiviral capabilities of CX T cells. Furthermore, we found the enrichment of Tconv Trms, and specifically the Th17 Trm subset, in the CX of BV^+^ individuals that may also serve as targets for HIV infection and replication. Confirmation of these findings and elaboration of molecular mechanisms may identify novel targets for immune interventions to reduce the risk of adverse health outcomes associated with BV, including increased risk of HIV infection.

## Methods

### Sex as a biological variable.

This study focuses on cervicovaginal immune alterations in the context of BV. Therefore, samples from individuals who were identified as female at birth were exclusively used.

### Participants, samples, and data collection.

Samples and data for this analysis came from the Kinga Study, which enrolled a total of 406 heterosexual Kenyan couples from October 2018 through December 2019 to evaluate how exposure to sexually transmitted infectious agents alters genital mucosal immune responses. Among these couples, 110 were HIV serodifferent, defined as a person living with HIV (PLWH) and their heterosexual HIV-exposed partner; the remaining 298 couples involved partners who were both HIV seronegative at enrollment. HIV-serodifferent couples were excluded if, prior to enrollment, the PLWH had initiated antiretroviral therapy (ART) with resulting suppressed HIV viral load, or the HIV-exposed partner had initiated tenofovir-based pre-exposure prophylaxis (PrEP). ART and PrEP were provided to enrolled PLWH or HIV-exposed partners, respectively, upon enrollment.

Seven samples were requested from all Kinga Study participants at enrollment and 6-month follow-up visits for evaluation of immune responses: CVT fluid via menstrual cup (Softcup; The Flex Company); two 3-mm VT tissue biopsies, with one cryopreserved for immunofluorescent imaging and one cryopreserved for flow cytometry; two 3-mm CX tissue biopsies processed in parallel to the VT biopsies; fractionated PBMCs; and serum were all collected. (Genital samples and CVT fluid collection were deferred if participants were actively menstruating, and participants were encouraged to return to clinic when not menstruating for collection of all sample types at the same time point.) Swabs for BV testing were collected before biopsies, and BV was assessed by Nugent score with 0–3 defined as normal flora, 4–6 defined as intermediate flora, and 7–10 classified as BV (101). Additional demographic, epidemiological, clinical, and sexual behavior data (including self-reported frequency of vaginal sex, condom use, and hormonal contraceptive use) were collected at all visits. Participants were screened for HIV and HSV-2 infection. Additional details related to clinical laboratory testing are described in Supplemental Methods.

To identify samples for the current cross-sectional analysis of the effects of BV on immune responses, we focused on 245 participants living without HIV who were identified as female at birth. These consisted of all 44 participants who may have been exposed to HIV by their enrolled heterosexual sexual partners, and 201 whose enrolled heterosexual sexual partner was without HIV and whose data could support a variety of analyses, including the analysis of BV-mediated immune responses. Immunofluorescent imaging was performed on enrollment samples from a subset who were HSV-2 seronegative and had HIV-uninfected partners. All immunological testing was performed blinded to participant exposure data.

### Analysis of tissue samples by high-parameter flow cytometry.

Biopsies were collected using baby Tischler forceps at either the lateral vaginal wall or the ectocervical os, placed in a cryovial containing fetal bovine serum at 4°C, and transported to the laboratory. In the lab, dimethylsulfoxide was added to the cryovial to a final concentration of 10%, and the biopsies were cryopreserved overnight at –80°C and transferred to liquid nitrogen for long-term storage as described by Hughes et al. (45). PBMCs were isolated using standard protocols.

Additional details related to sample collection, tissue processing for flow cytometry, and flow cytometry protocols and analyses are described in Supplemental Methods and Supplemental Table 4.

### Analysis of tissue samples by immunofluorescent microscopy.

Fresh-frozen CX and VT biopsies were sectioned and used for H&E staining and immunofluorescent staining. Tissue sections were imaged, and images were acquired and analyzed. Additional details related to sample collection, tissue staining, and tissue section image analysis are described in Supplemental Methods.

### Cytokine and chemokine sample collection and processing.

CVT fluid was collected via menstrual cup, and serum was collected in a Serum Separation Tube (SST) Vacutainer (BD). Levels of cytokines and chemokines from CVT fluid and serum samples were measured using the Human Cytokine Array/Chemokine Array 71-403 Plex Panel (Eve Technologies, HD71). Additional details related to CVT fluid and serum collection and processing and the soluble mediator assay are described in Supplemental Methods.

### Statistics.

For purposes of data analysis, BV^+^ was defined by Nugent score of 7–10. BV^–^ was defined as normal flora by Nugent score 0–3. For flow cytometry and soluble mediator analyses, to maximize the size of our BV^+^ group, we used enrollment visits of participants who were BV^+^ at enrollment, augmented with the 6-month visit from any additional participants who were BV^+^ at the 6-month exit visit. Thus, our BV^+^ group consisted of one time point per individual, but in some cases it was enrollment while for others it was at exit. Among the remaining individuals, enrollment visits for those with normal flora at enrollment served as the BV^–^ reference group. For immunofluorescent imaging, all specimens were from enrollment.

For both flow cytometry and soluble mediator data, results from VT and CX biopsy and CVT fluid samples were a priori adjusted for hormonal contraceptive use, HSV-2 serology, HIV exposure, and number of unprotected sex acts in the last 30 days, while results from PBMC and serum samples were a priori adjusted for hormonal contraceptive use only. In this exploratory work, we did not adjust *P* values for multiple testing. *P* values less than or equal to 0.05 were considered significant. Statistical analyses were conducted using R version 4.3.3. Additional details related to statistical analyses performed for laboratory tests are described in Supplemental Methods.

### Study approval.

All participants provided written informed consent using documents reviewed and approved by the University of Washington Human Subjects Division Institutional Review Board and the Scientific and Ethics Review Unit of the Kenya Medical Research Institute.

### Data availability.

The data for all analyses described in this article are available in the Supporting Data Values file. Additional data relevant to the Kinga Study may be made available upon reasonable request.

## Author contributions

FM, JBG, JLS, SCV, NP, ICT, and LW conducted the experiments. FM, ATT, JLS, AS, CM, and KKT analyzed data. MM provided reagents. JD and LKS contributed analysis methods. BHC, KN, NM, JRL, and JML designed the research study. FM, MCS, JRL, and JML wrote the first draft of the manuscript. All authors edited and approved the manuscript.

## Supplementary Material

Supplemental data

ICMJE disclosure forms

Supplemental table 1

Supplemental table 2

Supplemental table 3

Supporting data values

## Figures and Tables

**Table 1:**
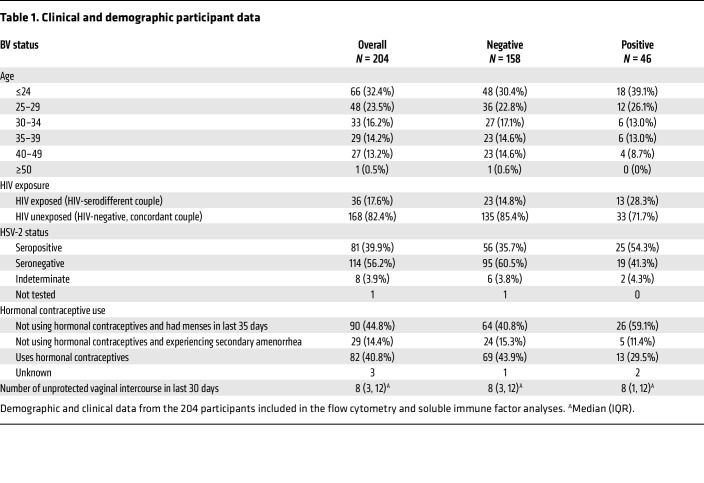
Clinical and demographic participant data

## Version 1 03/25/2025

In-Press Preview

## Version 2 05/15/2025

Electronic publication
