# Supplementary material for: Bacterial vaginosis associates with dysfunctional T cells and altered soluble immune factors in the cervicovaginal tract
Source: J Clin Invest. 2025 Mar 25;135(10):e184609. doi: 10.1172/JCI184609 (PMC12077898; doi:10.1172/JCI184609)
Supplement: Supplemental data [file jci-135-184609-s304.pdf]

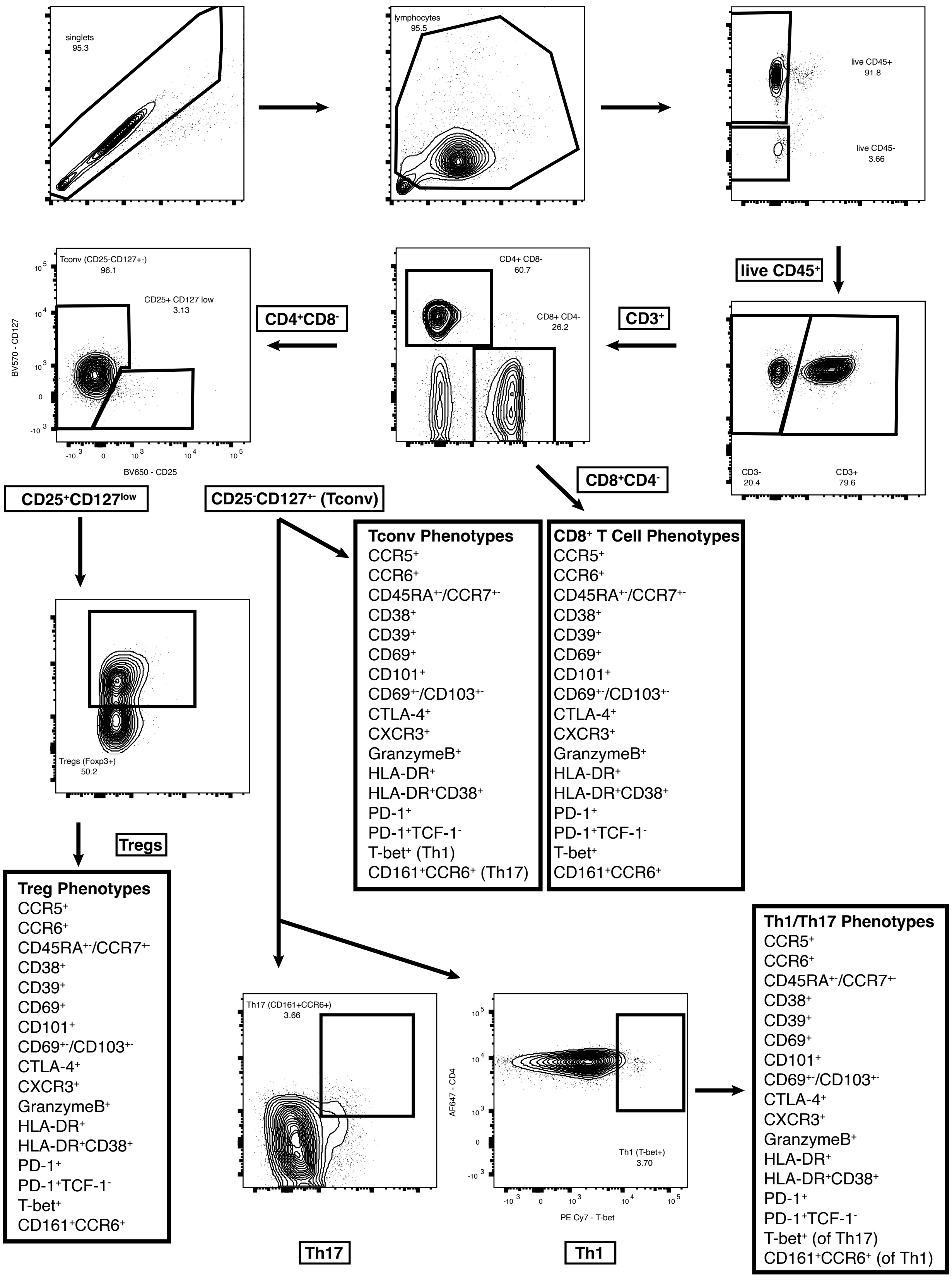

Supplemental Figure 1. Flow cytometry gating to determine phenotype expression on individual cells.
Cells were gated by forward scatter height (FSC-H) and forward scatter area (FSC-A) to filter out non-singlet cells. Then cells were gated by side scatter area (SSC-A) and forward scatter area (FSC-A) to isolate lymphocytes. Lymphocytes were analyzed by live/dead and CD45 expression. Live CD45^{+} cells were then analyzed for CD3 expression. CD3^{+} cells were then analyzed for CD4 and CD8 expression. CD8^{+}CD4^{-} were analyzed for the phenotypes listed. CD4^{+}CD8^{-} were analyzed for CD25 and CD127 expression. CD25^{-} cells were defined as conventional CD4^{+} T cells (Tconv). CD25^{+}CD127^{low} were analyzed for Foxp3 expression. Foxp3^{+} among the CD25^{+}CD127^{low} population were defined as regulatory T cells (Tregs). Tregs were analyzed for the phenotypes listed. Tconv cells were analyzed for the phenotypes listed. In addition, Tconv cells were analyzed for T-bet expression which defined Th1 cells, and CCR6^{+}CD161^{+} co-expression which defined Th17 cells. Th1 and Th17 cells were also analyzed for expression of the phenotypes listed. For each gating analysis, samples were only continued for further analysis if the parent gate had a minimum of 25 cells.

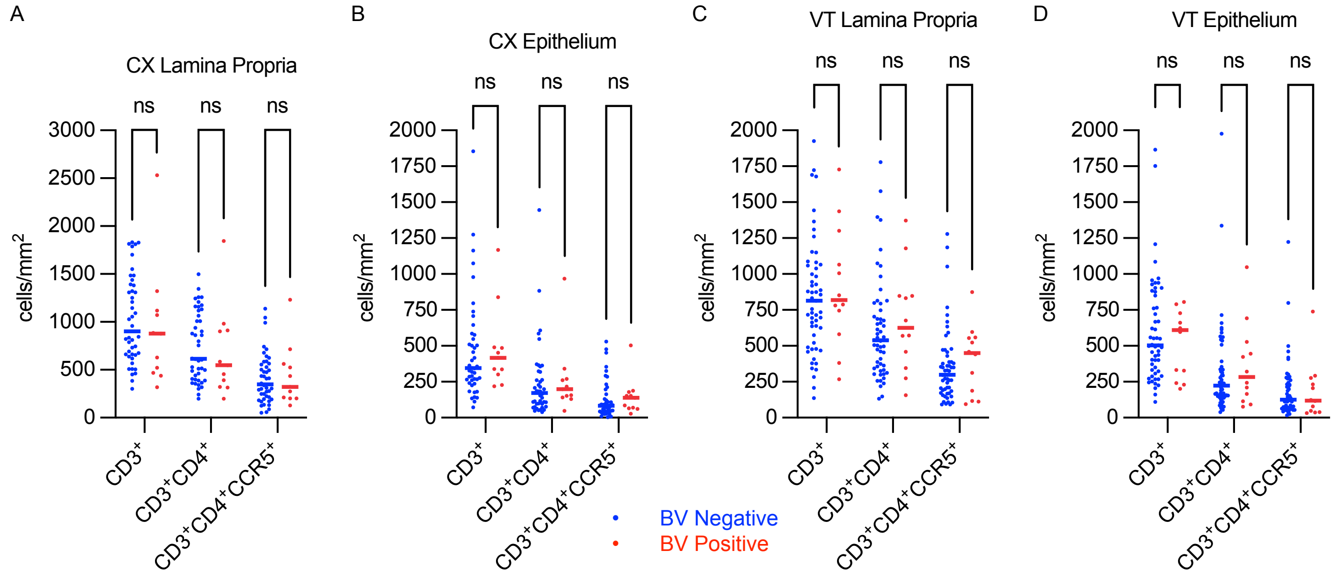

Supplemental Figure 2. HIV target cell abundance was not altered by BV across CX and VT tissue compartments.
Comparison of the cell density of CD3+ T cells, CD3+CD4+ T cells, or CD3+CD4+CD5+ HIV target cells between BV- and BV+ samples in the (A) CX lamina propria and (B) epithelium (CX BV- N=44, BV+ N=11) and (C) VT lamina propria and (D) epithelium (VT BV- N=55, BV+ N=12). Wilcoxon rank sum test was performed for each comparison shown. Comparisons with $p > 0.05$ labeled "ns" for not significant. Each dot represents a measurement from an individual sample. Each horizontal bar indicates the median for its respective group. Statistical analyses are available in Supplemental Table 2.

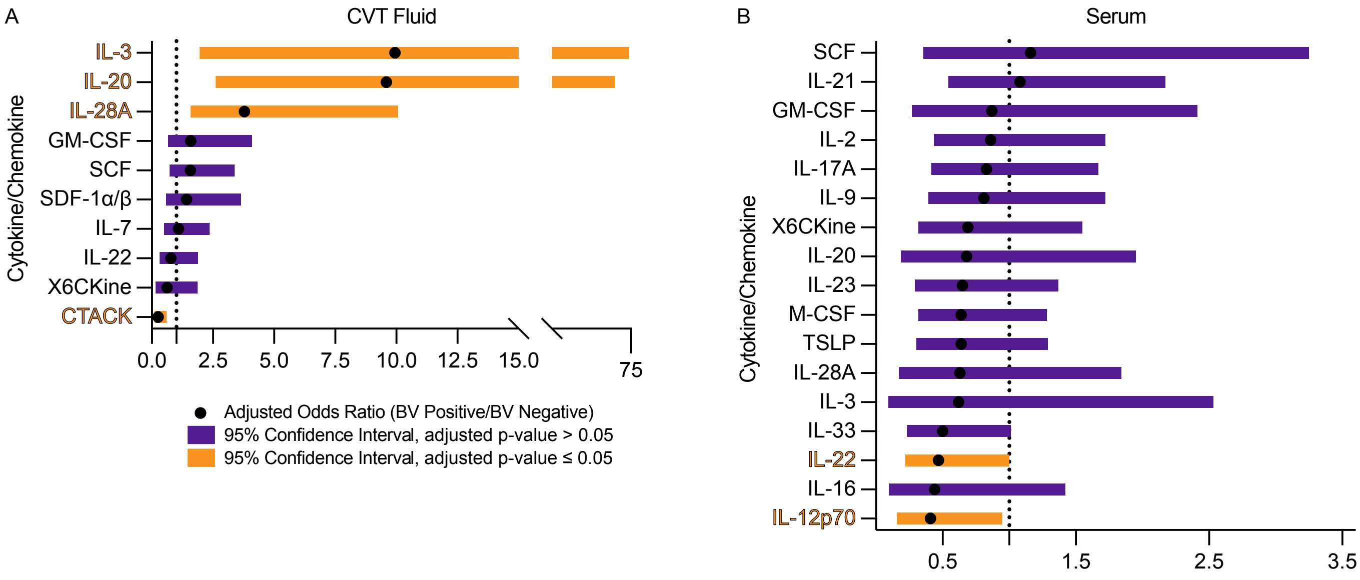

Supplemental Figure 3. BV was associated with altered detection of soluble factors in CVT fluid and serum.
Luminex was used to quantify cytokines and chemokines from N= 174 CVT fluid and N=193 serum samples. Adjusted odds ratio (BV positive/BV negative) for (A) CVT fluid cytokines/chemokines and (B) serum cytokines/chemokines that did not meet the criteria for quantification of cytokine/chemokine concentrations (fewer than 80% of samples were detectable). The adjusted 95% confidence interval is shown for all comparisons. Significant results when adjusted p ≤ 0.05 comparing BV- versus BV+ are colored in orange and non-significant differences (p > 0.05) comparing BV- versus BV+ are purple. Vertical dashed line at x=1 for reference. Serum comparisons were *a priori* adjusted for hormonal contraceptive use, and CVT fluid comparisons were *a priori* adjusted for hormonal contraceptive use, HSV-2 serology, HIV exposure, and semen exposure to reduce the effects of potential confounding variables on the analysis of BV-driven T cell alterations. Statistical analyses are available in Supplemental Table 3.

Supplemental Table 4. Antibodies used for high parameter flow cytometry on mucosal biopsies and PBMCs.

T cell panel used for phenotyping						
Channel	Fluorochrome	Antibody	Clone	Dilution	Company	Catalog #
UV395	BUV395	CD39	TU66	1:40	BD	742524
UV450	BUV450	Live/Dead		1:1000	Invitrogen	L23105A
UV500	BUV496	CD8	RPA-T8	1:500	BD	612943
UV570	BUV563	CCR7	2-L1-A	1:40	BD	749679
UV660	BUV661	CD3	UCHT1	1:200	BD	612964
UV730	BUV737	CD69	FN50	1:50	BD	612817
UV780	BUV805	CCR6	11A9	1:20	BD	749361
V510	BV510	CCR5	J418F1	1:166.7	BioLegend	359128
V570	BV570	CD127	A019-D5	1:83.3	BioLegend	351308
V610	BV605	PD-1	EH12.2H7	1:20	BioLegend	329924
V650	BV650	CD25	BC96	1:83.3	BioLegend	302634
V710	BV711	CD38	HIT2	1:40	BD	563965
V750	BV750	CD103	Ber-ACT8	1:250	BD	747099
V785	BV786	CD161	HP-3G10	1:83.3	BD	748281
B515	AF488	HLA-DR	1.243	1:83.3	BioLegend	307656
B660	BB660	SA		1:500	BD	6464295
B710	PerCpCy5.5	CD45	HI30	1:100	BD	564105
B780	BB790	CD101	V7.1	1:166.7	BD	Custom
G575	PE	TCF-1	C63D9	1:40	Cell Signaling	14456
G610	PE-CF594	Foxp3	236A/E7	1:20	BD	563955
G660	PE Cy5	CTLA4	BNI3	1:166.7	BD	561717
G780	PE Cy7	T-bet	4B10	1:20	Invitrogen	25-5825-82
R660	AF647	CD4	RPA-T4	1:40	BD	557707
R710	AF700	GranzymeB	GB11	1:83.3	BD	560213
R780	APC-H7	CD45RA	HI100	1:40	BD	560674
B660	Biotin	CXCR3	G025H7	1:80	BioLegend	353743

Supplemental Methods

Participants, samples, and data collection

Clinical Laboratory Testing: Laboratory testing at the enrollment and 6-month follow-up visits included HIV serology by Determine HIV 1/2 Rapid diagnostic Test (RDT) (Abbott Laboratories, Inc., Abbott Park, IL, USA) with confirmation of positive results with First Response RDT (Premier Medical Corporation Ltd., Kachigam, India), and further confirmation of RDT results using 4th generation HIV-1-2 Ag/Ab Murex EIA assay (DiaSorin, Inc., Kent, UK). Herpes simplex virus type 2 (HSV-2) serology was performed with HerpeSelect 2 ELISA IgG test (Focus Technologies, Inc., Cypress, CA) using an index value cut-off of ≥3.5 to improve test specificity (1-5).

Analysis of tissue samples by high-parameter flow cytometry

Sample collection: Peripheral blood mononuclear cells (PBMCs) were isolated from acid-citrate dextrose (ACD) whole blood by centrifugation and resuspension in Dulbecco's Phosphate Buffered Saline (DPBS). Cells were centrifuged over Ficoll-Histopaque (Sigma-Aldrich, Cat# 10771) at room temperature. The buffy coat was collected and washed twice in DPBS at 4°C before live cells were counted on a hemacytometer with trypan blue. Cells were pelleted and resuspended in 10% DMSO in fetal calf serum at 4°C at a concentration of either 5-7 x 10^6 cells/mL, or 10-15 x 10^6 cells/mL. Cryovials with 1 mL of these PBMC suspensions were placed in controlled rate freezing equipment (Mr. Frosty) and stored overnight at -80° C. Cells were subsequently transferred to liquid nitrogen for long term storage and shipped in liquid nitrogen dry shippers to University of Washington for further analysis.

Tissue Processing for Flow Cytometry: Cryopreserved PBMCs, ectocervical biopsies, or vaginal biopsies in liquid nitrogen were quickly thawed and transferred to 10% FBS (PBMCs) or 7.5% FBS (VT/CX biopsies) complete RPMI media (RP10 or RP7.5). PBMCs were spun down and resuspended in RP10. After sitting in warmed RP7.5 for 10 minutes, biopsies were incubated at 37 degrees C for 30 minutes with Collagenase II (Sigma-Aldrich, 700 U/mL) and DNase I (Sigma-Aldrich, 400U/mL) in RP7.5. Biopsies were then passed through a 100-mm cell strainer using a plunger to disrupt the tissue, washed, and resuspended in phosphate-buffered saline (PBS). PBMCs and cells isolated from tissues were stained immediately after processing for flow cytometry. Cryopreserved PBMCs from a healthy control donor (Seattle Area Control Cohort (SAC)) were used as a longitudinal technical control for all flow cytometry acquisitions (data not shown).

Flow Cytometry: Immediately following isolation, cells were incubated with UV Blue Live/Dead reagent (Supplemental Table 4) in PBS for 30 mins at room temperature. After washing, cells were then stained with biotinylated CXCR3 (Supplemental Table 4) in 0.5% FACS buffer for 20 mins at room temperature. Cells were washed and then stained extracellularly with antibodies (Supplemental Table 4) diluted in 0.5% FACS buffer and brilliant staining buffer (BD Biosciences, Cat# 563794) for 20 mins at room temp. Cells were fixed with Foxp3 Transcription Factor Fixation/Permeabilization buffer (ThermoFisher, Cat# 00-5521-00) for 30 mins at room temp. Cells were washed and then stained intracellularly with antibodies diluted in 1X Permeabilization Buffer (ThermoFisher, Cat# 00-8333-56) for 30 mins at room temp. Cells were then resuspended in 200 ml 0.5% FACS buffer and stored at 4 degrees until ready for use. Antibodies were titrated and used at optimal dilution. Staining was performed in 5-ml polystyrene tubes (Falcon, Cat# 352054). Analysis was performed using Flowjo software and

we required at least 25 cells for analysis of phenotypes associated with the parent cell type and further downstream gating and analysis (Supplemental Figure 1).

Analysis of tissue samples by immunofluorescent microscopy

Sample collection: Biopsies were collected using baby Tischler forceps at the lateral vaginal wall and the ectocervical os, embedded in optimal cutting temperature (OCT) compound and cryopreserved on dry ice.

Laboratory Analysis: Fresh frozen tissue biopsies in OCT compound were sectioned 8 μm thick using a cryostat and placed onto frosted microscope slides. Serial sections were used for H&E staining and immunofluorescent (IF) staining. H&E-stained slides were used to identify lamina propria and epithelium and to evaluate tissue integrity to ensure tissue sections met the criteria for IF analysis (intact lamina propria and epithelium).

Tissue sections for IF staining were fixed in acetone at -20°C for five minutes, dried for thirty minutes at room temperature, and rehydrated in tris-buffered saline with 0.05% tween (TBST). Slides were quenched using 3% H_2O_2 for twenty minutes at room temperature, rinsed with TBST, and endogenous binding sites were blocked using 1:10 fish gelatin blocking agent (Biotium, Cat# 22010) diluted in TBST for one hour. Unconjugated mouse anti-human CCR5 antibody (clone MC-5 (6) provided by M. Mack) at optimal dilution (0.3 μg/ml) was added for one hour, washed with TBST, and then goat anti-mouse antibody conjugated with horseradish peroxidase (polyclonal, Invitrogen, Cat# B40961) was added for thirty minutes. After washing with TBST, a solution of Tris Buffer (100mM, MilliporeSigma, Cat# 648315), optimally diluted tyramide (AF488, Invitrogen, Cat# B40953, diluted 1:100), and H_2O_2 (diluted 1:67,000) was then used for immunofluorescence and signal amplification. Slides were washed with TBST and

a combination of optimally diluted, directly conjugated anti-CD3 (AF594, Clone UCHT1, R&D Systems, Cat# FAB100T, diluted 1:50) and anti-CD4 (AF647, Clone RPA-T4, BioLegend, Cat# 300520, diluted 1:25) antibodies were added for incubation overnight in the 4°C fridge. Slides were then rinsed in TBST and stained with DAPI (Invitrogen, Cat# D1306, diluted 2.5 ng/ml) for five minutes. Slides were washed with PBS, saturated briefly for ten seconds in ammonium acetate, saturated for ten minutes in copper sulfate, and then saturated again briefly for ten seconds in ammonium acetate. Slides were thoroughly rinsed in dH_2O, dried, mounted with Prolong Gold (Invitrogen, Cat# P36930) and a cover slip, and allowed to set for 24 hours.

Tissue sections were imaged within 48 hours of staining using a 20x/0.8 Pan-APOCHROOMAT air objective on a Zeiss Axio Imager Z2 microscope as part of a TissueFAXS system (TissueGnostics; Vienna, Austria) equipped with an X-cite 120Q lamp (Excelitas), and with DAPI (49000 ET), EGFP (49002 ET), Texas Red (49008 ET), and Cy5 (490006 ET) filter sets. Images were acquired using an Orca-Flash 4.0 camera (Hamamatsu) with TissueFAXS 7.1 software (TissueGnostics).

Tissue section image analysis

We quantified the density of $CD3^+$, $CD3^+CD4^+$, and $CD3^+CD4^+CCR5^+$ cells in the epithelium and lamina propria of CX and VT sections from individuals with or without BV. Images acquired using the TissueFAXS system were exported as multi-channel 16-bit tiff files of stitched regions. Cell segmentation, region of interest (ROI) selection (lamina propria vs epithelium), marker intensity thresholding, and quantification of densities were estimated using a custom graphical user interface (GUI) developed in MATLAB (R2022b). Nucleus segmentation was performed by finding the regional maxima of the grayscale DAPI signal, followed by

morphological thickening constrained by the binary DAPI mask. Threshold intensities for the markers of interest were set manually through the GUI. The boundary between the lamina propria and epithelium regions was segmented based on nuclear density and further adjusted manually through the GUI. Finally, for each region (lamina propria and epithelium), the number of single-, double-, and triple-positive cells, as well as the density (number of cells per mm^2), were extracted. Codes and an extended description of the GUI can be found at github.com/FredHutch/Kinga_Study_BV_MacLean.

Cytokine and chemokine sample collection, and processing

Cervicovaginal fluid: The menstrual cup was inserted into the vaginal canal beneath the cervix at the beginning of the study visit and remained in place for a minimum of 15 and a maximum of 60 minutes while other visit procedures were being conducted. Sample collection was deferred if the participant was menstruating. The participant was encouraged to move around while the menstrual cup was in place. Once removed, the menstrual cup and all CVT fluid contents were placed in a 50 mL sterile collection tube and transported to the lab on ice. CVT fluids were collected in the 50 mL collection tube by centrifuging at 1500 rpm (320xg) for 10 min at 4°C. The mucous and fluids were gently mixed and 200-300 µl was aliquoted with a 1000 mL pipet tip into each cryovial and stored at -80°C.

Serum: Whole blood was collected in an SST vacutainer, allowed to clot for less than 2 hours, and centrifuged at 1300g for 15 minutes. Serum was aliquoted and stored at -80ºC.

Soluble mediator assays: Serum and menstrual cup (CVT fluid) aliquots were shipped on dry ice to Eve Technologies (Calgary, Alberta, Canada). All samples were measured upon the first thaw. CVT fluid samples were weighed and diluted in PBS at a ratio of 1g sample to 1mL

PBS. CVT fluid samples were vortexed for 2 minutes and up to 500 μl was loaded into 0.2 μm low-bind spin filters (MilliporeSigma, Cat# CWLS01S03) and centrifuged at ~14,000 x g for 15 mins to remove the mucus from the samples prior to analysis. Levels of cytokines and chemokines from CVT fluid and serum samples were measured using the Human Cytokine Array/Chemokine Array 71-403 Plex Panel (Eve Technologies, HD71).

Statistics

Cell densities of $CD3^{+}$, $CD3^{+}CD4^{+}$, and $CD3^{+}CD4^{+}CCR5^{+}$ cells in tissue sections were compared between individuals with versus without BV using the Wilcoxon rank sum test. To estimate adjusted differences in the percentage of T-cells with specified markers from flow cytometry data by BV status, we used rank-based regression, a nonparametric method robust to outliers (7). To investigate associations of soluble mediators in serum and CVT fluid samples with BV status, we first determined whether, for each mediator, at least 80% of the specimens generated a quantifiable level (vs out of quantifiable range or missing for another reason). If at least 80% of results were quantifiable, we imputed out of range values by randomly selecting a value between the lowest observed value and half the lowest observed value (if out of range low), or by selecting the largest observed value (if out of range high). We then compared $\log_{10}$-transformed levels of the mediator from individuals in BV+ vs BV- groups using a t-test and estimated differences in mean log cytokine concentrations from both serum and CVT fluid samples using linear regression. If <80% of the levels for a mediator were quantifiable, we categorized the mediator as detected vs not detected and estimated the odds ratio for the effect of BV on mediator detection using logistic regression. For both flow cytometry and soluble mediator data, results from VT and CX biopsy and CVT fluid samples were *a priori* adjusted for hormonal contraceptive use (yes, no and menstruating, no and amenorrheal), HSV-2 serology

(positive, negative, indeterminate), HIV exposure (HIV status of sexual partner), and number of unprotected sex acts in the last thirty days (continuous), while results from PBMC samples were *a priori* adjusted for hormonal contraceptive use (yes, no and menstruating, no and amenorrheal), only. In this exploratory work, we did not adjust p-values for multiple testing. P-values of less than or equal to 0.05 were considered significant. Statistical analyses were conducted using R version 4.3.3.

Supplemental Acknowledgments

Kinga Study Team:

University of Washington: International Clinical Research Center

Jairam R Lingappa (co-Principal Investigator and Protocol Chair), Justice Quame-Amaglo (study coordinator), Harald Haugen, Elena Rechkina (Laboratory Director), Daphne Hamilton, Matthew Ikuma, Marie Bauer, Zarna Marfatia, Kathy Thomas, Corinne Mar, Adino Tesfahun Tsegaye, Ayumi Saito

Fred Hutchinson Cancer Center:

Jennifer Lund (co-Principal Investigator), Sarah Vick, Finn MacLean, Jessica Graham, Jessica Swarts, Nicole Potchen, Irene Cruz Talavera, Lakshmi Warrier, Laura Pattacini, Paula Culver

Kenya Medical Research Institute: Thika Partners in Health Research and Development (and Jomo Kenyatta University [JKUAT])

Nelly R. Mugo (Site Principal Investigator), Kenneth Ngure (Site Investigator), Catherine Kiptinness (site coordinator), Bhavna H. Chohan (Site Laboratory Director), Nina Akelo, Stephen Gakuo, Elizabeth Irungu, Marion Kiguoya, Edith Kimani, Eric Koome, Solomon Maina, Linet Makena, Sarah Mbaire, Murugi Micheni, Peter Michira, Peter Mogere, Richard Momanyi, Edwin Mugo, Caroline Senoga, Mary Kibatha, Jelioth Muthoni, Euticus Mwangi, Philip Mwangi, Margaret Mwangi, Charles Mwangi, Stanley Mugambi Ndwiga, Peter Mwenda, Grace Ndung'u, Faith Njagi, Zakaria Njau, Irene Njeru, John Njoroge, Esther Njoroge, John Okumu,

Lynda Oluoch, Judith Achieng Omungo, Snaida Ongachi, Dennis Wanyonyi, Everlyne Okong'o, Agnes Ndirangu

References

1. Golden MR, Ashley-Morrow R, Swenson P, Hogrefe WR, Handsfield HH, and Wald A. Herpes simplex virus type 2 (HSV-2) Western blot confirmatory testing among men testing positive for HSV-2 using the focus enzyme-linked immunosorbent assay in a sexually transmitted disease clinic. *Sex Transm Dis.* 2005;32(12):771-7.
2. Laeyendecker O, Henson C, Gray RH, Nguyen RH, Horne BJ, Wawer MJ, et al. Performance of a commercial, type-specific enzyme-linked immunosorbent assay for detection of herpes simplex virus type 2-specific antibodies in Ugandans. *J Clin Microbiol.* 2004;42(4):1794-6.
3. Gamiel JL, Tobian AA, Laeyendecker OB, Reynolds SJ, Morrow RA, Serwadda D, et al. Improved performance of enzyme-linked immunosorbent assays and the effect of human immunodeficiency virus coinfection on the serologic detection of herpes simplex virus type 2 in Rakai, Uganda. *Clin Vaccine Immunol.* 2008;15(5):888-90.
4. Lingappa J, Nakku-Joloba E, Magaret A, Friedrich D, Dragavon J, Kambugu F, et al. Sensitivity and specificity of herpes simplex virus-2 serological assays among HIV-infected and uninfected urban Ugandans. *Int J STD AIDS.* 2010;21(9):611-6.
5. Mujugira A, Morrow RA, Celum C, Lingappa J, Delany-Moretlwe S, Fife KH, et al. Performance of the Focus HerpeSelect-2 enzyme immunoassay for the detection of herpes simplex virus type 2 antibodies in seven African countries. *Sex Transm Infect.* 2011;87(3):238-41.

6. Segerer S, Mac KM, Regele H, Kerjaschki D, and Schlondorff D. Expression of the C-C chemokine receptor 5 in human kidney diseases. *Kidney Int.* 1999;56(1):52-64.

7. McKean JW, and Hettmansperger TP. A Robust Analysis of the General Linear Model Based on One Step R- Estimates. *Biometrika.* 1978;65(3):571-9.
